# Dynamic Control Balancing Cell Proliferation and Inflammation is Crucial for an Effective Immune Response to Malaria

**DOI:** 10.3389/fmolb.2021.800721

**Published:** 2022-02-15

**Authors:** Anuj Gupta, Mary R. Galinski, Eberhard O. Voit

**Affiliations:** ^1^ The Wallace H. Coulter Department of Biomedical Engineering, Georgia Institute of Technology and Emory University, Atlanta, GA, United States; ^2^ Emory Vaccine Center, Yerkes National Primate Research Center, Department of Medicine, Division of Infectious Diseases, Emory University, Atlanta, GA, United States

**Keywords:** *Plasmodium knowlesi*, *Macaca fascicularis*, *Macaca mulatta*, kynurenine metabolism, tryptophan metabolism, AhR signaling, antigenic variation, surface antigens

## Abstract

Malaria has a complex pathology with varying manifestations and symptoms, effects on host tissues, and different degrees of severity and ultimate outcome, depending on the causative *Plasmodium* pathogen and host species. Previously, we compared the peripheral blood transcriptomes of two macaque species (*Macaca mulatta* and *Macaca fascicularis*) in response to acute primary infection by *Plasmodium knowlesi*. Although these two species are very closely related, the infection in *M. mulatta* is fatal, unless aggressively treated, whereas *M. fascicularis* develops a chronic, but tolerable infection in the blood. As a reason for this stark difference, our analysis suggests delayed pathogen detection in *M. mulatta* followed by extended inflammation that eventually overwhelms this monkey’s immune response. By contrast, the natural host *M. fascicularis* detects the pathogen earlier and controls the inflammation. Additionally, *M. fascicularis* limits cell proliferation pathways during the log phase of infection, presumably in an attempt to control inflammation. Subsequent cell proliferation suggests a cell-mediated adaptive immune response. Here, we focus on molecular mechanisms underlying the key differences in the host and parasite responses and their coordination. *SICAvar* Type 1 surface antigens are highly correlated with pattern recognition receptor signaling and important inflammatory genes for both hosts. Analysis of pathogen detection pathways reveals a similar signaling mechanism, but with important differences in the glutamate G-protein coupled receptor (GPCR) signaling pathway. Furthermore, differences in inflammasome assembly processes suggests an important role of S100 proteins in balancing inflammation and cell proliferation. Both differences point to the importance of Ca^2+^ homeostasis in inflammation. Additionally, the kynurenine-to-tryptophan ratio, a known inflammatory biomarker, emphasizes higher inflammation in *M. mulatta* during log phase. Transcriptomics-aided metabolic modeling provides a functional method for evaluating these changes and understanding downstream changes in NAD metabolism and aryl hydrocarbon receptor (AhR) signaling, with enhanced NAD metabolism in *M. fascicularis* and stronger AhR signaling in *M. mulatta*. AhR signaling controls important immune genes like IL6, IFNγ and IDO1. However, direct changes due to AhR signaling could not be established due to complicated regulatory feedback mechanisms associated with the AhR repressor (AhRR). A complete understanding of the exact dynamics of the immune response is difficult to achieve. Nonetheless, our comparative analysis provides clear suggestions of processes that underlie an effective immune response. Thus, our study identifies multiple points of intervention that are apparently responsible for a balanced and effective immune response and thereby paves the way toward future immune strategies for treating malaria.

##  Highlights

• *Macaca mulatta* and *Macaca fascicularis* are closely related macaque species that respond very differently to infection with the malaria pathogen *Plasmodium knowlesi*.
• Early detection, sensing of the pathogen with associated signaling, and balance between inflammation and cell proliferation are the most important differences in the immune response of the two hosts.
• Pathogen surface antigens of *SICAvar* Type 1 are most highly correlated with host immune and pathogen sensing mechanisms.
• Pre-infection differences in neutrophils and naïve CD4+ T cells result in differences in Ca^2+^ homeostasis, which ultimately balances inflammation and cell proliferation during the expansion log phase of the parasitemia.
• Dysregulation of ribosomal protein assembly in *Macaca fascicularis* causes p53-dependent growth arrest, which is essential for balancing the immune response and inflammation.
• Tryptophan metabolism and its key control gene KMO balance downstream energy metabolism and inflammation pathways through NAD^+^ metabolism and AhR signaling, hence playing an important role in the balance of cell proliferation, immune response and inflammation.

## Introduction

Malaria is one of the world’s deadliest infectious diseases, with an estimated 229 million cases and 409,000 deaths reported in 2019 (World malaria report 2020, 2020). It is caused by parasites of the genus *Plasmodium*. Ethical reasons render investigations of molecular host-responses in malaria difficult in humans, because treatment of patients is obligatory as soon as they are diagnosed. Rodent malaria models have been widely used to expand our understanding of these infections, but present drawbacks due to major differences in human and mouse or rat genetics and physiology. By contrast, nonhuman primates (NHPs) are much closer to humans, and the clinical presentation of malaria and consequent immune responses are quite similar in humans and macaques (Coatney et al., 1971; Aikawa et al., 1992; Coatney et al., 2003; Gardner and Luciw, 2008; Craig et al., 2012; Joyner et al., 2015; Pasini et al., 2018).

Here, we contrast the drastically different responses of two evolutionarily close macaque species (Morales and Melnick, 1998; Tosi et al., 2000), the kra monkey (*Macaca fascicularis*, Mf) and the rhesus monkey (*Macaca mulatta*, Mm), to infection with the same pathogen, *Plasmodium knowlesi*. These two hosts are the most-studied model NHPs and their infections with various pathogens is studied as it is often comparable to those in humans (Van Binnendijk et al., 1995; El Mubarak et al., 2007a; Baroncelli et al., 2008; Sasseville and Mansfield, 2010; Salguero et al., 2021). They are evolutionarily so close (3.7 MYA) (Hedges et al., 2015) that one might expect a similar immune response to a common pathogen. Long before *P. knowlesi* became a zoonotic concern, Knowles and Gupta (1932) identified Mf as a natural host for *P. knowlesi* infection. Since then, numerous infection experiments have demonstrated that the two macaques respond very differently to infection (Garcia et al., 2004; El Mubarak et al., 2007b; Maiello et al., 2018; Lu et al., 2020). Whereas Mf develops a chronic infection that it tolerates relatively well, the *P. knowlesi* infection in Mm is fatal, unless the monkey is subjected to aggressive treatment. This outcome is somewhat surprising, as it is widely accepted that Mm typically outcompetes other macaque species, including Mf. A likely explanation is that Mf co-evolved with *P. knowlesi* within a large geographical area of Southeast Asia, whereas the distribution of Mm overlaps with that of *P. knowlesi* only slightly (Street et al., 2007; Singh and Daneshvar, 2013; Moyes et al., 2014; Gupta et al., 2021). Studies analyzing these differences have begun to show that Mf generally launches a more effective immune response (Waag et al., 1999; El Mubarak et al., 2007a; Pinski et al., 2021). However, a better understanding of the control of the biological programs that differentiate the immune responses is of utmost importance, because it will not only offer insights into the details of these responses but may also point to molecular targets that might lead to improved malaria treatments for humans.

In a recent transcriptomics study (Gupta et al., 2021), we analyzed the gene programs with which Mm and Mf respond to a *P. knowlesi* infection, initiated with infectious sporozoites. This comparative analysis revealed numerous transcriptomic similarities, but also notable differences. In particular, Mf, but not Mm, apparently detects this pathogen as early as the liver phase of the infection, prior to the parasite infecting the blood, and this correspondingly activates beneficial signaling pathways early on. Later in the infection, significant differences arise in each monkey’s immune responses, which in Mm lead to extended inflammatory activities and prolonged inflammation. By contrast, Mf contains the infection and controls inflammation by undergoing a transcriptional makeover toward cell proliferation that accompanies its recovery.

The goal of the present study is to shed light on some of the molecular mechanisms governing the different gene programs and thus the ultimate fates of the two macaque species. In particular, the study identifies and quantifies: differences in the detection of the pathogen, associated differences in the immune response, differences in cell proliferation that directly affect the immune response and indirectly inflammation and, finally, differences in pathways that regulate inflammation.

The detection of malarial parasites by the host immune system is driven by parasite-encoded surface proteins, including among others the Schizont-Infected Cell Agglutination (SICA) variant proteins (Lapp et al., 2009) that are expressed from the *SICAvar* gene family (al-Khedery et al., 1999; Wahlgren et al., 1999; Pain et al., 2008; Lapp et al., 2018; Galinski et al., 2018). The antigenicity and variability provided by these various proteins stimulates the production of antibody repertoires and immunogenicity that have been widely studied in the context of vaccine development (Ferreira et al., 2004; Ouattara et al., 2015; Rénia and Goh, 2016; França et al., 2017). The *Plasmodium* pathogen multiplies within infected red blood cells (iRBCs) and once matured these cells burst releasing new merozoite progeny that infect other RBCs. This process generates pathogen- and damage-associated molecular patterns (PAMPs and DAMPs), which in turn stimulate various pattern recognition receptor (PRR) signaling pathways in macrophages, monocytes, neutrophils and dendritic cells, and execute various immune mechanisms via protein kinase cascades (Alberts, 2002; Schroder and Tschopp, 2010). Co-expression analysis has been shown to be instrumental in determining these host-pathogen interactions (Lee et al., 2018). The neutrophils and macrophages not only target foreign content for phagocytosis but also trigger the inflammatory and adaptive immune response. Our previous analysis showed much stronger inflammation in Mm compared to Mf, which launches extensive measures to control cell proliferation. The balance between these pro- and anti-inflammatory mechanisms appears to be the key to resilience, and a deeper understanding of the underlying mechanisms is therefore of utmost importance (Cicchese et al., 2018). The energy-intensive nature of these processes makes metabolic processes like glycolysis and tryptophan (Trp) metabolism close accomplices in regulating the overall physiological dynamics. Furthermore, the feedback loop of Trp metabolism and Aryl hydrocarbon Receptor (AhR) signaling in controlling inflammatory cytokines is essential for this balance.

The complete dynamics of the entire immune response is obviously difficult to comprehend in full detail, as this response is systemic and involves uncounted facets, some evident but others subtle. Thus, while our comparative analysis clearly cannot convey a complete picture of all chains of causes and effects governing the responses by the two macaque species, it offers a first glimpse into some of the same and some of the differentiating processes evoked by the two monkey species. The study thereby opens a new avenue toward potential future strategies of immune-based malaria treatments and provides multiple promising candidates for interventions targeting a balanced and effective immune response.

## Results

Our analysis is based on data that were obtained with an experimental design (Supplementary Figure S18) recently detailed in (Peterson et al., 2021) and (Gupta et al., 2021). In this longitudinal study of *P. knowlesi* infections in Mm and Mf, peripheral blood and bone marrow samples were collected at various time points (TPs), including baseline (before infection), pre-patent (TP3 or 3 days post inoculation; dpi), log-phase (TP4 or eight dpi) and peak-phase (TP5 or 10 dpi). The first signs of parasitemia were observed six dpi, and the infection increased exponentially thereafter. The Mm subjects were euthanized by 10 dpi, at the time parasitemias were escalating to dangerous levels, to carry out necropsies and characterize the infected tissues. We previously observed that Mf shows very early signs of parasite detection by three dpi (Gupta et al., 2021). Even though the immune response was found to be similar between the hosts during the log-phase of the blood infection, Mf was found to switch its response near peak infection towards cell proliferation, which we concluded is a sign of recovery. In the current study we address these and other findings to shed additional light on the molecular mechanisms governing these processes.

### Correlated Nonhuman Primate Host and *P. Knowlesi* Transcripts Suggest Common Signaling Mechanisms and the Expression of Key Pathogenic Proteins, Including SICA Antigens

**FIGURE 1:**
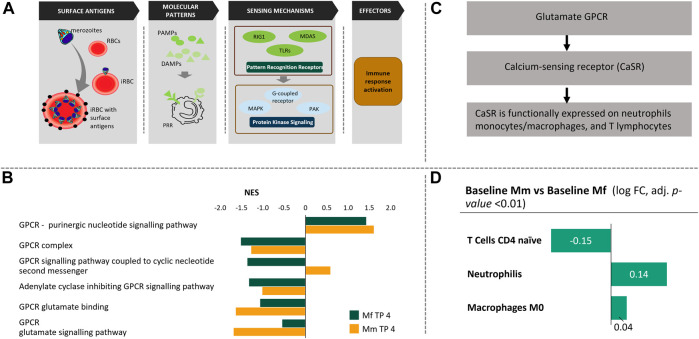
Chain of events during the blood phase of *P. knowlesi* infection. **(A)** Pathogen and RBCs: Once released from the liver into the blood stream, merozoites invade uninfected RBCs leading to Infected RBCs (iRBC) with exposed surface antigens (schizont‐infected cell agglutination antigens—SICA; black). The iRBCs are partially eliminated by macrophages, a process that triggers the production of pathogen/danger-associated molecular patterns (PAMPs/DAMPs). These PAMPs/DAMPs are sensed by other immune cells through Pathogen Sensing Mechanisms (PRR signaling), which activate various protein kinase signaling pathways. These signaling pathways are responsible for immune response activation that is mediated through various leukocytes. **(B)** Among several protein kinase signaling mechanisms, GPCR signaling pathways are enriched in both hosts. While purinergic nucleotide GPCRs are similarly enriched, glutamate GPCRs are noticeably different in the two hosts. **(C)** Glutamate GPCRs are responsible for calcium sensing and functionally expressed in neutrophils, monocytes, macrophages and T cells. **(D)** Comparative cell population deconvolution at baseline (i.e., before infection) shows these populations to be different, which might reflect an innate difference in Ca^2+^ signaling in the two host species.

It is to be expected that a mammalian host senses the presence of a parasite based on the detection of pathogenic macromolecules or signals from infected erythrocytes, which trigger signaling pathways in the host that in turn control the gene programs governing a systemic immune response (Figure 1A). In this section, we analyze the sensing-signaling process by means of co-expression networks, functional annotation, and logistic regression analysis.

#### Co-Expression Networks of Host and Parasite Genes

Genes with similar functionality often have correlated expression profiles, which may be identified using co-expression network analysis (Fuller et al., 2007). We adapted this approach by combining both host and pathogen transcripts in a weighted correlation network analysis (WGCNA) (Langfelder and Horvath, 2008) in order to identify modules of host and pathogen genes that act in synchrony. We refer to these modules based on their “hub genes.” Specifically, the analysis resulted in three types of modules: (A) Host modules consisting exclusively of host genes; (B) Host majority modules with both host and parasite genes, but with a majority of host genes; and (C) *P. knowlesi* majority modules with both host and parasite genes, but with a majority of *P. knowlesi* genes (Supplementary Figure S2).

Genes involved with essential functions form well-defined modules (Supplementary Table S1A). It is not surprising that most of the differentially expressed genes (DEGs) during the parasitemic log phase belong to the TOP1 module (immune response), followed by C1D and ATAD3A modules (with insignificant functional annotation) for both hosts. It is worth noting that the NF2 (tRNA metabolic process), SNRPD2 (ribosomal assembly) and RACGAP1 (mitotic cell cycle) modules are most highly correlated with the TOP1 module (Pearson correlation between eigengenes with *p* < 0.01, corrected for false discovery rate (FDR)), suggesting close orchestration between these essential functions. The interactions of host and pathogen genes are most evident through interactions between Type C modules and Type A or Type B modules. Significantly high correlations between host and *P. knowlesi* genes (Supplementary Table S1B) are found in modules ATAD3A (Type B) and PKNOH_S08507800 (Type C). Interestingly, 23 out of the 26 highly correlated *P. knowlesi* transcripts belong to Schizont-Infected Cell Agglutination variant antigen (*SICAvar*) Type 1 genes (al-Khedery et al., 1999; Pain et al., 2008; Lapp et al., 2018) (Supplementary Table S1C). The corresponding SICA variant antigens, which are expressed on the surface of infected erythrocytes (Howard et al., 1983) and associated with virulence (Galinski et al., 2018), show high correlations with several important host genes, including IL10, ELK4 and HSPA6. This suggests that *SICAvar* Type 1 transcripts play a role in regulating inflammation in the host, for example, directly through IL10 expression and indirectly by regulating stress signals through HSPA6 expression.

#### Parasite Gene Expression Affecting Host Co-Expression Modules

In order to create more functionally robust host modules, we used WGCNA with all host samples (including Baseline and TP3), while excluding *P. knowlesi* genes (Supplementary Table S2). Logistic regression followed by functional enrichment identified key modules changing during infection in both host species. The defense response module FBXO6, and modules GFRA2 and RASGEF1A (with insignificant functional annotation), were the most different during the log phase. Modules that were different included RPS19 (SRP-dependent co-translational protein targeting to membrane) and NR1H3 (cell activation involved in immune response). Integrating module membership data with host-pathogen transcripts correlation data (see Section on *co-expression networks*) highlighted *Plasmodium* proteins that affected each module. Most noticeable are *SICAvar* Type 1 (Pain et al., 2008; Lapp et al., 2018), Trp-rich antigen (Wang et al., 2015) and KIR-like proteins (Pain et al., 2008) affecting modules RPS19 and NR1H3, which differentiated the two hosts. Additionally, high correlation of hemoglobin complex module EPB42 with several pathogenic ribosomal proteins suggests a possible mechanism for digesting hemoglobin as an essential nutrition source for the pathogen (Counihan et al., 2021). Host specific WGCNA revealed similar results with *SICAvar* Type 1 being most correlated to host immune modules (Supplementary Notes).

#### Pattern Recognition Receptor Signaling

The co-expression network analysis is able to detect important host-pathogen relationships that appear to be crucial for the two hosts. In order to focus on detection of the pathogen by a host, we modified the analysis to create a customized module of PRR signaling related genes, which allowed us to identify *Plasmodium* proteins that interact with their products. The most positively correlated pathogen proteins include KIR-like protein (Pain et al., 2008) and thioredoxin-like protein (Wang et al., 2018a; Yindom et al., 2012). Among the genes that negatively co-express with the PRR module are *SICAvar* Type II, AP-1 complex subunit sigma and histones H2A/H2B. The host genes most highly correlated with *P. knowlesi* genes include pathogen detection genes like IFIT3, PLA2G4C, MX1, OASL, DDX60, OAS2, RSAD2, MX2, DHX58, IFIH1, STAT1, FAS and TLR4 (for functional annotations (Huang et al., 2009), see Supplementary Table S3A). As reported before (Gupta et al., 2021), it is worth noting that most of these signaling genes are upregulated in Mf at TP3, which further supports the hypothesis of earlier pathogen detection in Mf than in Mm.

For many mammalian hosts, the PRR signaling pathway has been credited for detecting PAMPs or DAMPs, DNAs, and other large molecules (Figure 1A) (Mogensen, 2009; Roh and Sohn, 2018; Amarante-Mendes et al., 2018). In our case, the activity of this pathway is consistent with expression of PRR-related genes in both hosts during the appropriate infection time points (TPs; Supplementary Table S3B). Here we concentrate on the log phase of infection because this phase is associated with the most similar features between the two hosts, and any observed differences might highlight critical processes. It is interesting to note that many of the PRR signaling genes are in fact different between Mm and Mf. This difference implies that even though the more general PRR signaling pathway is activated in response to the detected pathogen, the specifics of the pathway operation are apparently different, which could be due either to the detected pathogenic content or the interpretation of the signaling event by the host’s immune responses. The major genes differentiating the specifics of PRR signaling include TLR5, NLRP6, TNIP3, SLC15A4, SLC15A3, CD36 and CD300A. We had reported enriched pathways before, but the specific differences are not as easy to deduce (Supplementary Table S4). Such differences are evident in subsets of the TLR signaling cascade, especially in TICAM1/RIP1 mediated IKK complex recruitment. Higher expression of corresponding ubiquitination genes (UBE2D1, UBA52, RPS27A and BIRC2) in Mm suggests activation of NFκB (Festjens et al., 2007), which might be responsible for stronger inflammation.

These differences between hosts are carried forward toward responses by networks of protein kinases. We examined enrichment of several protein kinase cascades including mitogen-activated protein kinase (MAPK), G protein-coupled receptor (GPCR) systems and p21-activated cascades. Both host species exhibit higher activation of atypical cytokine activated MAPK4/6 signaling involving PAK (p21 activated kinases) (De la Mota-Peynado et al., 2011; Déléris et al., 2011) in comparison to the typical stress activated p38/MAPK signaling pathway (Supplementary Figure S3A). Major differences in protein kinase activities are associated with higher inhibition activity in Mm, which is probably due to peptidyl tyrosine dephosphorylation. Although not fully understood, this pathway has been implicated in both pro- and anti-cell proliferation roles (Kostenko et al., 2012) and might be responsible for downstream differences in p53 and HSP27 related cell cycle activity. Differential regulation of protein kinase C activity (Supplementary Figure S3B) might explain these differences (Saha et al., 2014).

Probably the most notable difference between the two species is observed in their GPCR signaling (Figure 1B). Although both species show similar positive enrichment of purinergic nucleotide GPCR signaling, differences in glutamate GPCR signaling highlight their differences in inflammation (Figure 1B). Purinergic nucleotide GPCR activity explains the upregulation of purine metabolism in malaria and points to a potential role of macrophages (Barberá-Cremades et al., 2016). Macrophage production is significantly upregulated in both hosts during log phase (Supplementary Tables S5A,B), with the same direction of fold change as the purinergic nucleotide GPCR signaling. In contrast to these similarities, glutamate GPCRs, which are Ca^2+^ sensing receptors, show clear differences between the two hosts in both binding and subsequent signaling pathways, thus suggesting downstream implications of calcium homeostasis (Figure 1C). As discussed later in the section *discussing the effects of inflammation*, Ca^2+^ homeostasis plays a crucial role in inflammation. Ca^2+^ sensing glutamate GPCRs are functionally expressed on neutrophils, monocytes, macrophages and T lymphocytes. An innate difference between the two hosts is their difference in these cell populations at baseline (Figure 1D, Supplementary Table S5C). Some of these differences have been corroborated in the literature (Koo et al., 2019).

### Ribosomal Proteins Control p53 Pathway

**FIGURE 2:**
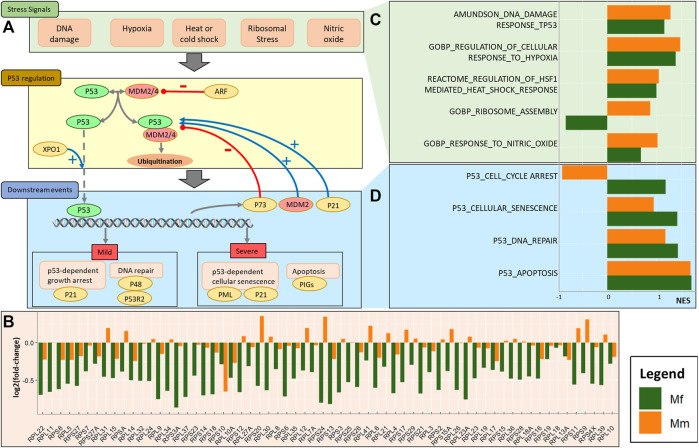
Involvement of the p53 pathway. **(A)** Schematic of cause, regulation, and effect of p53 pathway activation during the log phase of a malarial infection. The overall process includes key stress signals that engage the p53 regulation pathway and result in regulation of downstream events. **(B)** Barplot for log2 fold-changes in the expression of ribosomal proteins between TP4 and baseline, comparing the two hosts. **(C)** Barplot for normalized enrichment scores (NESs) of stress signals involved in p53 activation. **(D)** Barplot for normalized enrichment scores of p53 related downstream events.

Our previous work (Gupta et al., 2021) had suggested control over the p53 pathway during the log phase of the infection as a crucial difference between the immune responses of the two host species. Binding of p53 to its target response element leads to the expression of a multitude of genes with a spectrum of functions, including cell cycle growth arrest, DNA repair, cellular senescence and apoptosis (Haupt et al., 2002) (Figure 2). An active p53 pathway also protects cells against reactive oxygen species (ROS) through antioxidant genes like TP53INP1 (Sablina et al., 2005) (Supplementary Figure S4). Indeed, the early response of this pathway in Mf (at TP4) might be crucial in saving cells from apoptosis via PIG3 (TP53I3 gene) (Lee et al., 2010). Co-expression analysis, discussed in the earlier section on *parasite gene expression*, revealed several closely regulated modules controlling DNA binding, the mitochondrial envelope, and the mitotic cell cycle, all of which are more strongly enriched in Mf (Supplementary Table S2).

As observed in the previous section, certain MAPK signaling mechanisms potentially regulate the p53 pathway. Generically, the p53 pathway is operational in the presence of molecular stresses and depends on their severity as well as other factors. Cellular stress signals that activate a p53 response include hypoxia, DNA damage, ribosomal and oxidative stresses, among others (Joerger and Fersht, 2016). These stresses are, of course, not independent of each other and manifest in an interrelated manner. In the case of a *P. knowlesi* infection, this interdependence can be seen in the enrichment of associated genes. In particular, the enrichment analysis demonstrates that ribosomal stress is a differentiating factor between the two host species, with stress caused by substantial downregulation of the ribosomal assembly complex in Mf (Figure 2C). This downregulation is achieved through the activity of various RNA polymerases (Supplementary Figure S5), and the significant downregulation of PLOR1C, POLR2E, POLR2A, POLR2J and POLR2L in Mf at TP4 suggests that these genes might be crucial for the control of p53.

The downregulation of associated ribosomal proteins (RPs) in Mf at TP4 (Figure 2B, Supplementary Figure S6) is indicative of alterations in ribosomal biosynthesis that results in unassembled RPs and 5S rRNA, which binds to the p53 inhibitors MDM2 and MDM4 and thereby prevents p53 degradation (Golomb et al., 2014; Haupt et al., 2019). As a consequence, p53 facilitates translation from its mRNA internal ribosome entry site (IRES). Indeed, the co-expression network analysis reveals high correlation of MDM4 with *SICAvar* Type 1 transcripts, which suggests direct control that might be crucial in this regulation.

As a consequence of RP downregulation, the p53 pathway in Mf is upregulated, which is reflected in higher levels of enrichment. However, the less pronounced changes in cell cycle arrest and DNA repair appear to be the strongest differentiating factors between the two species. The important genes involved in these processes include CDKN1A (p21), E2F7, PML and MDM2 (upregulated) and TP73 (downregulated).

Another facet of p53 pathway activation and control is provided by the transcription factor HSF1 (heat shock factor 1). Dysregulation of ribosomal biosynthesis processes leads to proteotoxic stress and a balance between these processes must be maintained (Albert et al., 2019). In comparison to Mf, Mm has higher ribosomal biosynthesis and senses higher proteotoxic stress during the log phase of infection, and these processes are further increased near the peak (Supplementary Figure S7). Of note in this context is the differential expression of chaperone-mediated protein folding genes—HSPA1A, HSPA8, DNAJB1 and FKBP4. This expression results in upregulation of HSF1 target genes in Mm. p53 has been shown to form multi-chaperone complexes with HSPA1, DNAJB1 or HSPA8, while FKBP4 is essential for its transport to the nucleus (Toma-Jonik et al., 2019). Among the apoptotic targets, ATF3 enrichment at both TP4 and TP5 in Mm highlights differences with Mf.

Finally, the control over cell proliferation in Mf is eased near the peak, which leads to upregulation of adaptive immune cells and, in particular CD4 memory activated and follicular helper cells (Supplementary Table S7F), both of which enhance the adaptive immune response by supporting B cells and CD8 T cells (MacLeod et al., 2009; Crotty, 2014).

### Effects of Inflammation on Immune Response and Cell Proliferation

Control of cell proliferation in Mf during the log phase of infection constitutes a stark contrast to the elevated inflammatory response in Mm. This difference can be seen clearly in the enrichment of several inflammatory pathways, elevated expression of inflammatory genes, inflammasomes and inflammatory biomarkers like the kynurenine (Kyn)/Trp ratio (see Supplementary Figures S8–S10 and next section). Co-expression analysis revealed that most of the inflammatory genes are part of the innate immune module (FBX06 module). Not surprisingly, this module is most significantly changed (according to logistic regression) in both hosts during log phase. Although the fold-change for this module is similar between the hosts, the lower adjusted *p* value (*q*) and higher log-odds (*B*) suggest a stronger role of this module in the innate immune response in Mf (*q* < 3e-17/*B* > 32) in comparison to Mm (*q* < 1e-10/*B* > 17). This module further highlights the differences between the hosts near peak infection as Mm (*q* < 7e-7/*B* > 8) maintains its immune response while Mf (*q* < 4e-5/*B* > 1) does not. The neutrophil activation and intracellular vesicle transport (FYB) module is most highly correlated to the innate immune module (FBX06). Also, worth noting is that both these modules are negatively correlated to ribosomal biosynthesis and localization modules RPS19, ZNF395, CHD6 and RASGEF1A. This finding implies an important, sustained balance between immune related inflammation and control over the cell cycle.

Several similarities in inflammation gene sets are found between the hosts, especially with respect to an LPS-like inflammatory response, which probably is a symptom of the NLRP3 inflammasome (Supplementary Figure S8A). This phenomenon might be attributed to significant upregulation of monocytes and monocyte-derived pro-inflammatory M1 macrophages (Supplementary Tables S7A,B), which are the first-line cells expressing inflammasome genes (Awad et al., 2017) (Supplementary Figure S10). Important differences are detected in the inflammatory response cytokine production and an antigenic stimulus (Supplementary Figure S8B). Even though the positive regulation of these functions is similarly enriched in the two hosts, as seen in the important genes NOD2, GPX1 and IL12B, the negative regulation shows a distinct and opposing enrichment. The main distinguishing genes include IL10, NLRP6 and ABCD1.

The two hosts show similar enrichment of the chronic inflammatory response; however, Mm has a higher acute inflammatory response (Supplementary Figure S8B), reaffirming the stronger inflammation in Mm during log phase. The chronic inflammation changes near the peak and is mostly driven by crucial genes like IL10, IDO1, TNF, TNFAIP3 and CXCL13 (Supplementary Figure S9).

**FIGURE 3:**
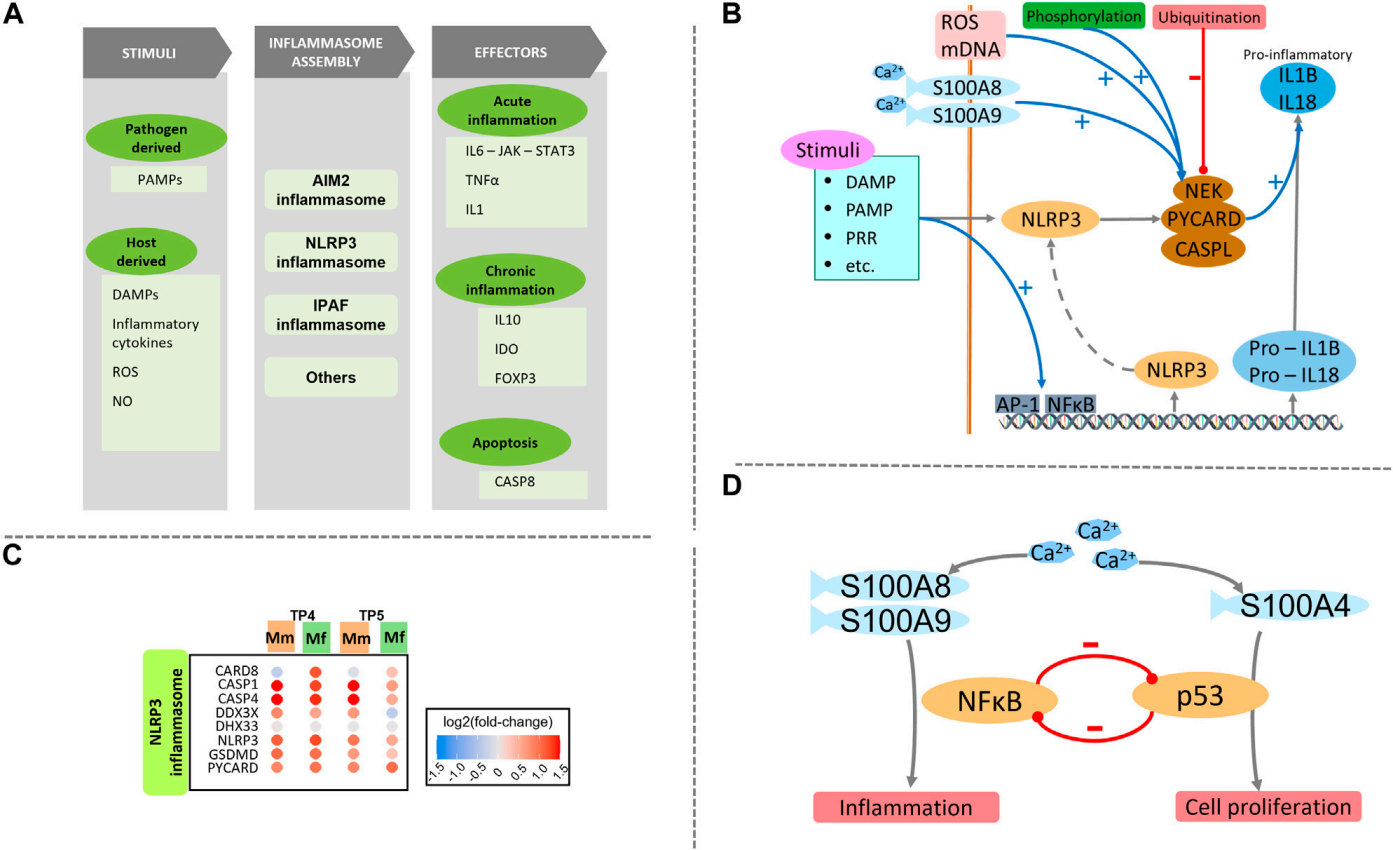
**(A)** Schematic of the inflammasome assembly process. Various host and pathogen derived stimuli are responsible for initiation of the inflammasome assembly process. This process is very closely regulated by various signals and processes including ROS and Ca^2+^. Several effectors execute important processes like inflammation and apoptosis. Different effectors are activated during acute and chronic phases. **(B)** Detailed schematic of NLRP3 inflammasome signaling including the initiation and regulation of the assembly process, followed by pro-inflammatory effectors. **(C)** Heatmap of genes involved with NLRP3 inflammasome assembly process are similarly enriched between the two hosts. **(D)** Balance and cross-regulation of p53 and NFκB showing importance of Ca^2+^ homeostasis.

Exploration of innate immune components of inflammation reveals a crucial difference in S100 proteins (Figure 3). Ca^2+^ sensing S100 proteins have a wide range of functionality that includes cell apoptosis, proliferation and inflammation (Figure 3B) (Fox and Man, 2019). Differential upregulation of S100A8, S100A9, S100A16 and S100P in Mm suggests a potential role of Ca^2+^ in inflammation (Wang et al., 2018b), while upregulation of S100A4, S100A2 and S100A3 in Mf suggests possible regulation of p53 (Figure 3D) (Pan et al., 2018; Boye and Mælandsmo, 2010). Since neutrophils release S100A8/A9 during inflammation, their differential expression mediates Ca^2+^ signaling, which positively regulates NLRP3 inflammasome assembly and pro-inflammatory activity of NFκB (Figures 3B,C) (Wang et al., 2018c; Xia et al., 2018). This inflammatory activity is further exacerbated by master regulator DDX3X (Fox and Man, 2019). Further enrichment of processes specifically associated with innate and adaptive immune processes reveals an interesting pattern that succinctly differentiates the responses of the two hosts. Namely, TLR4 signaling is stimulated by Ca^2+^ via S100 proteins (S100A8 and S100A9), which enhances the inflammatory activity of the NLRP3 inflammasome. These inflammatory pathways are responsible for IFNβ regulation and IL6 production. This finding directly complements earlier findings of differential Ca^2+^ glutamate GPCR activity (Section *PRR signaling*), which directly affects the inflammasome assembly.

Further exploration of differences in immunological signatures reveals several important similarities and differences between the two hosts (Supplementary Figure S11). Both hosts show significant enrichment towards FOXP3+ CD4^+^ naïve T-reg cells (GSE37533 (Cipolletta et al., 2012), GSE42021 (Toker et al., 2013)), with gene sets pointing to the strongest enrichment of a thymic T-reg subset of intermediate maturation, CD24^int^. A related important difference appears between the two hosts: Mf has a higher mature (CD24^low^) subset while Mm has a higher immature (CD24^hi^) subset. Both hosts have enriched naïve B cells (GSE42724 (Covens et al., 2013)), even though this change could not be confirmed by deconvolution analysis. Differences can be seen in gene sets derived from IL6 and IL10 stimulation as well. Taken together, these signatures suggest downregulation of key genes in Mf, which is not observed in Mm. Important genes that seem to be regulating this process in both Mm and Mf include IL6, IL6R, TGFB3, IL23A, IL10 and SOCS3.

Although not conclusive, pre-infection state differences in cell populations point to eventual differences in the immune response. For instance, at baseline, Mm has significantly more naïve CD4^+^ T cells while Mf has higher levels of neutrophils (Koo et al., 2019) (Figure 1D, Supplementary Table S5C). Although there is no significant relative difference in cell populations during the log phase of infection between the hosts (Supplementary Table S5E), these initial pre-infection differences persist (Supplementary Table S5D) and may be a key differentiating factor in the immune response.

### Changes in Tryptophan Metabolism Suggests Higher NAD^+^ Metabolism

**FIGURE 4:**
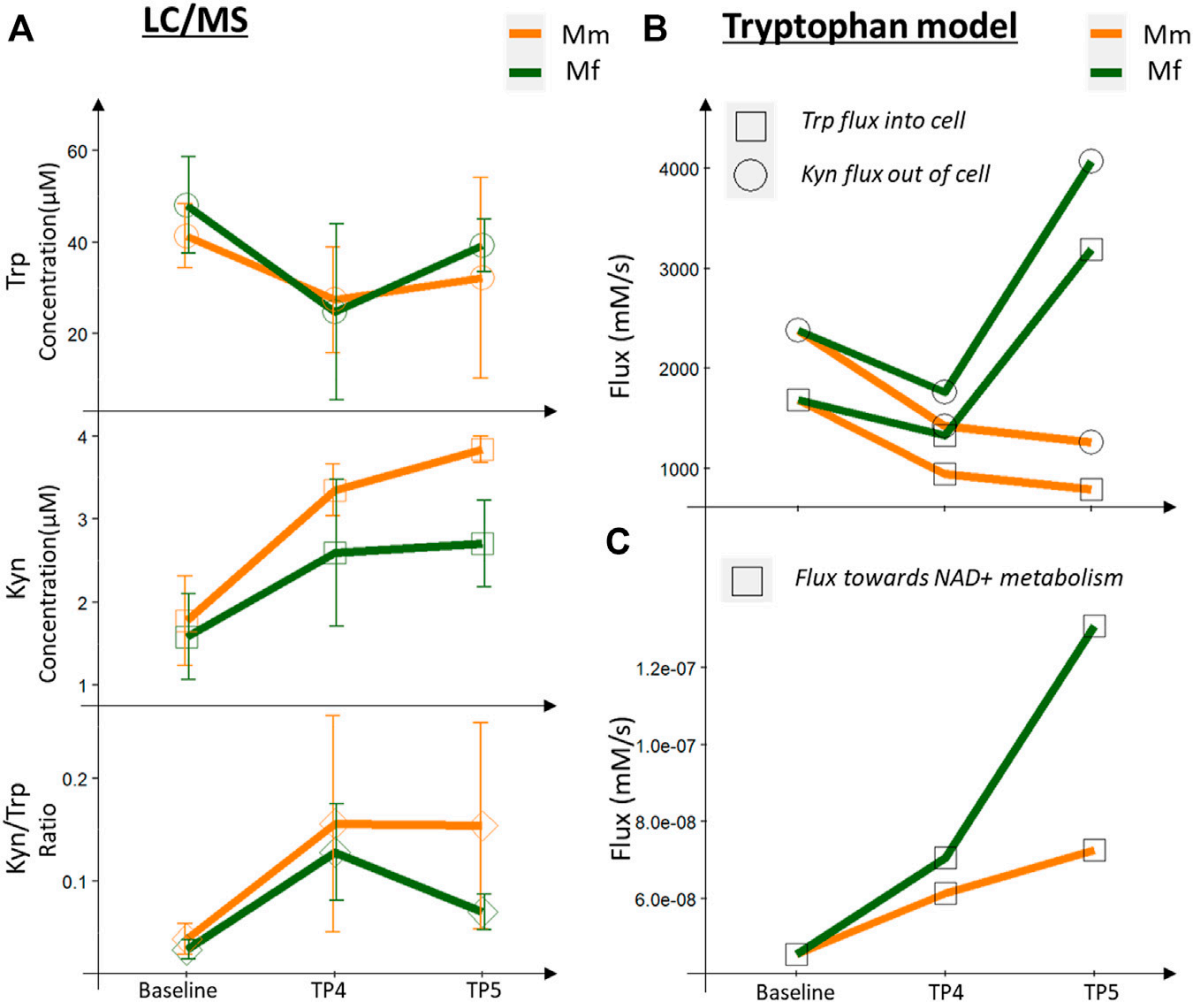
Metabolomics (LC-MS measurements) and model predictions for tryptophan metabolism. **(A)** LC/MS measurements for Trp, Kyn and the Kyn/Trp ratio across infection timepoints, comparing Mm and Mf. **(B)** Fluxes predicted by Trp model for Trp consumed and Kyn produced by blood cells. **(C)** Flux towards NAD metabolism predicted by the Trp model.

Metabolomic and transcriptomic analysis of the Mf and Mm hosts revealed prominent differences in the expression of genes associated with Trp metabolism at TP4 and TP5 (Figure 4). Trp metabolism can coarsely be divided into pathways responsible for serotonin and melatonin, Nicotinamide Adenine Dinucleotide (NAD^+^) and Kyn synthesis. Serotonin and related compounds are not of interest in the present context, and their concentrations in blood are very low. NAD^+^ metabolism plays a crucial role in cellular energy regulation as well as the handling of ROS. The Kyn pathway is responsible for the biosynthesis of several metabolites that play key roles in immunomodulation and inflammation.

Both hosts had lower Trp and higher Kyn levels in the peripheral blood during the log and peak phases of infection in comparison with baseline levels (TP1 and TP2), as observed previously with *P. coatneyi* infection of rhesus macaques (Cordy et al., 2019; Colvin and Joice Cordy, 2020). Of special note here, however, is that the Kyn/Trp ratio, a known inflammatory biomarker, is reduced in Mf near peak infection, whereas it remains at the same level as during the log phase in Mm (Figure 4).

IFNγ signaling is responsible for upregulating the expression of IDO (Taylor and Feng, 1991; Sarkar et al., 2007; Banzola et al., 2018), which converts Trp into Kyn (Figure 4A). Even though IFNγ signaling is upregulated in both hosts, a higher degree of signaling in Mf near the log and peak phases results in higher IDO expression, which thereby leads to a higher conversion of Trp to Kyn in Mf than in Mm (Figure 4C). One might expect that this increased activity should lead to a higher level of Kyn. Yet, we observe lower levels of Kyn and a lower Kyn/Trp ratio in Mf (Figure 4A), which however is easily explained by the increased activity of the subsequent enzymes KYNU and KMO in the Kyn utilization pathway, which ultimately lead to higher NAD^+^ biosynthesis and immunomodulatory activities. In addition, earlier downregulation of AhR and AADAT in Mf suggests potential differences in AhR signaling (see next section).

In order to elucidate the role of Trp and Kyn levels in the blood and then understand changes in Trp-Kyn metabolism in white blood cells (WBCs) during the infection, we adopted existing metabolic models of Trp metabolism in brain and liver (Stavrum et al., 2013) and adapted them to reflect Trp metabolism in WBCs. With this adapted model we can clearly differentiate Trp-Kyn metabolism in blood from brain and liver (Supplementary Figure S12). The model confirms that over 90% of Trp in whole blood is channeled toward Kyn through the activity of IDO, compared to tryptophan-2,3-dioxygenase (TDO) in the liver model. Kyn itself is the substrate for different reactions, and flux control analysis (Fell, 1992; Fell, 2007) reveals that KMO is the most important control point for Kyn utilization. Extension of the blood model during the log phase of the infection shows for both host species that the transport of both Trp and Kyn through the cell membrane is lowered in comparison to the baseline (Figure 4). This finding is interesting as it potentially leads to serum levels of Trp and Kyn in both hosts that change significantly during this phase and lead to a higher Kyn/Trp ratio (Figure 4). At the same time, these increases are accompanied by a major reshuffling of fluxes, which affects the metabolite concentrations inside the cells.

Specifically, Trp can be metabolized through six reactions, among which the pathway toward Kyn is most important, based on relative fluxes (Figure 4A). Indeed, if the Trp concentration inside the cells is decreased, the effluxes out of the Trp pool are also decreased, with the notable exception of the Kyn pathway, which receives essentially a normal influx from Trp. This flux is important, because the pathway later leads to the formation of quinolinic acid, which is a precursor of NAD^+^ and thus affects energy metabolism and redox handling. At this juncture, the differential expression of KMO becomes even more important as a control point for Trp metabolism: here, it causes a higher flux in Mf toward NAD^+^ synthesis (Figure 4C). This enhanced flux from Trp to Kyn is compensated in both species by decreased fluxes from Trp toward protein synthesis and serotonin production (Supplementary Figure S13).

Near the peak of infection, the differences in the two hosts are particularly pronounced, with Trp and Kyn transported through the cell membrane at higher rates in Mf than in Mm. As a result, the fluxes through the Kyn pathway are higher in Mf (Supplementary Figure S14). At the same time, the Kyn/Trp ratio is lower in Mf during this phase of the infection, presumably due to the enhanced activity of KMO (Figure 4A). It is also worth noting that the higher flux toward NAD^+^ metabolism persists in Mf (Figure 4C). Furthermore, the concentrations of other Kyn compounds remain high, and these are potential ligands of the AhR (see next section), which ultimately serves as a transcription factor for numerous genes (Figure 4B).

Among the other effluxes out of Trp, the indole-pyruvate and tryptamine pathways are also responsible for AhR activation (Hubbard et al., 2015; Roager and Licht, 2018) (next section). Trp is incorporated into proteins via tryptophanyl-tRNA synthestases (WARS proteins), a process that directly links Trp sensing to p53 activation (Yu et al., 2021). Changes in these fluxes during infection further show the central role of Trp metabolism (Supplementary Figures S16A,B).

### Aryl Hydrocarbon Receptor Signaling and the Role of the Aryl Hydrocarbon Receptor Repressor in Controlling Aryl Hydrocarbon Receptor and HIF1A Signaling

AhR belongs to the basic helix–loop–helix-PER-ARNT-SIM (bHLH-PAS) superfamily of transcription factors where multiple other members interact with each other and therefore affect each other’s functionality. Prominent members include AhRR, ARNT and HIF1α (Supplementary Figure S15).

Most of the biologically active intermediates of the Kyn pathway, as well as several other compounds, can act as ligands for AhR (Murray et al., 2014; Gutiérrez-Vázquez and Quintana, 2018) (Figure 4B, Supplementary Table S6), which makes this receptor a central control point for multiple physiological changes, *e.g.*, in heme degradation, hypoxia and Trp metabolism. Once a ligand binds, AhR can form a complex with the nuclear transporter ARNT, which is translocated to the nucleus. Once in the nucleus, the AhR-ARNT complex binds to the ARE promoter region of numerous genes.

Different Kyn derivatives may act as ligands for AhR, and their dynamics differentiates the Mf and Mm hosts during infection. Additionally, IL4I1 activity leading to indole-pyruvate derivatives from Trp synthesis activates AhR (Zhang et al., 2020). Similarly, multiple other ligands have been associated with AhR activity, and some of these may constitute further differences between the two host responses (Supplementary Figures S16C–F). For instance, *Plasmodium*’s consumption of hemoglobin releases heme, which is metabolized (Supplementary Figure S17). Certain AhR ligands that are derived from heme metabolism (Kapitulnik and Gonzalez, 1993; Phelan et al., 1998) may point to additional differences between the two hosts.

Another level of control of AhR signaling occurs through the competition between AhR, AhRR and HIF1α for ARNT and thus for transport into the nucleus and binding to their corresponding response elements. Given the molecular similarity of the competitors, it is not surprising that most of the downstream genes are simultaneous targets of both the AhR-ARNT and the HIF1α-ARNT complexes (Supplementary Table S7A).

Exploring these targets through an enrichment analysis shows that both complexes act quite similarly during log phase (Supplementary Figure S18B, TP4). Yet, several differences emerge near the peak of infection (Supplementary Figure S18B, TP5). The most pronounced differences emerge with respect to higher upregulation of AhR targets and HIF1α targets in Mf, while targets of the AhR repressor AhRR are downregulated in Mf. It appears that the relative hypoxia stress is quite different between the two hosts (Supplementary Figure S19), but it is unclear how the balance is achieved between these complexes and their corresponding genes.

To shed light on the interference among these complexes, many of which share numerous common targets, we calculated enrichment of each subset of these targets (Supplementary Figure S18). Specifically, we divided the targets into three major groups (AhR targets, HIF1α targets, and AhR and HIF1α targets) and compared them with and without the AhRR binding site to account for repressor activity (Supplementary Figure S18A).

The effect of AhRR on AhR targets is quite clear in Mf, with lower enrichment of targets at both TP4 and TP5, as opposed to almost no effect in Mm. Corresponding effects of AhRR on HIF1α targets are not easily identified. At TP4, HIF1α targets with the AhRR binding site are more enriched than without AhRR. At TP5, AhRR containing HIF1α targets are enriched more in Mm and less in Mf.

As there are multiple levels of regulation, it is difficult to predict the activity of these targets without further experimentation. However, one may try to elucidate the specific functionality of these targets by identifying the key genes along with their functional annotation. The transcription factor complexes in question are associated with a wide range of genes with diverse functionality (Figure 4). Functional annotation of AhR and HIF1α targets shows their involvement in key process like the p53 pathway, heme metabolism, cell cycle related pathways, and immune related IFNγ and NFκB pathways (Supplementary Table S7B). The complex nature of this response makes it difficult to elucidate the specifics and differences during a *P. knowlesi* infection, but the activity of individual genes suggests potential outcomes. Their roles in immune and inflammatory processes are evident in the activity of genes like OASL, STAT3, IRF5, IL6, DDIT4, NRF2, REL, and LAG3. These IFNγ signaling genes create a positive feedback loop, because IFNγ directly regulates IDO expression, which leads to enhanced levels of the AhR ligand Kyn. The control over cell proliferation is evident in the operation of p53 and other cell cycle related genes like MXD1, FOS, BCL6, GADD45A, and CREBRF. Another possible contributor with respect to malarial infection is heme metabolism with target genes including CCND3, BLVRB, and KLF1.

## Discussion and Conclusion

Malaria has haunted mankind throughout its history. Even after several decades of active research, malaria continues to be a severe global health concern with over 400,000 fatalities and about 3.2 billion people at risk annually. Among the six species of *Plasmodium* known to cause malaria in humans, *P. knowlesi* has become recognized as a major zoonosis in Southeast Asia (Cox-Singh, 2012; Barber et al., 2017; Zaw and Lin, 2019; Raja et al., 2020). A *P. knowlesi* infection in humans may range from mild to severe, with 6–10% of the cases considered severe (Singh et al., 2004; Daneshvar et al., 2009). A deeper knowledge of the details of *P. knowlesi* infections can be expected to provide a crucial basis for understanding the immune responses in general and for comparing resilient and severe malarial responses in particular. As a zoonotic species, *P. knowlesi* has the advantage that it can be studied in different NHP species (Pasini et al., 2018; Peterson et al., 2021). Among these NHP models, *Macaca mulatta* (Mm) and *M. fascicularis* (Mf) provide unique advantages specifically for comparing *P. knowlesi* infections with different disease progression. Namely, even though Mm and Mf are evolutionarily very close, Mm, once infected, suffers from increasing parasitemia, which is in almost all cases fatal if not treated, whereas Mf controls parasitemia and escapes death without treatment (Knowles and Gupta, 1932; Napier and Campbell, 1932; Peterson et al., 2021). These dramatic differences provide unparalleled opportunities to study the details of host physiology and immune responses in the context of host-parasite interactions and explore mechanisms of resilience in human malaria, and to potentially relate the findings to other diseases that may also show drastically different possible outcomes.

In previous work, we had established crucial differences in the transcriptomics of the two hosts that ultimately determines the outcome in terms of susceptibility and resilience (Gupta et al., 2021). As also noted in the clinical assessment by Peterson *et al.* (Peterson et al., 2021), transcriptomics analysis showed that Mf detects the pathogen earlier than Mm, and even though both host species mount a similar immune response, Mf starts controlling inflammation as early as the log phase of infection (Gupta et al., 2021). Subsequently, Mf switches the immune response towards cell proliferation pathways, which presumably aids recovery (Gupta et al., 2021). The current analysis explores the key findings further and explains the molecular functions that determine the mild or fateful outcome. Interestingly as well, early detection of the parasites by the Mf animals is also consistent with a rise in temperature in this species immediately upon patency, by seven dpi (Peterson et al., 2021).

The current results show consequential differences in signaling mechanisms beginning with the early detection of the presence of *P. knowlesi* pathogens by Mf. Once the merozoites invade the RBCs, they transform the iRBC and express different antigenic forms of surface molecules in an attempt to escape the immune response (Brown and Brown, 1965; Howard et al., 1983; Biggs et al., 1991). Specifically, antigenic variation of *P. knowlesi* SICA proteins is a main factor responsible for chronicity in Mm (Brown and Brown, 1965) (reviewed in Galinski et al. (2018)). Moreover, expression of *SICAvar* genes in *P. coatneyi* have been shown to change as chronic rhesus monkey infections are established, also suggesting a role for metabolites in regulating these changes (Cordy et al., 2019). Our correlation analysis of host and pathogen transcripts sheds light on possibly involved *SICAvar* Type 1 genes (al-Khedery et al., 1999; Pain et al., 2008; Lapp et al., 2018) along with correlated host genes. The specific correlations of individual transcripts from this large pathogen gene family—with 136 *SICAvar* members (Lapp et al., 2018)—could shed light on its transcripts and their variable gene expression, which may trigger different antibody responses. Additionally, correlations with host genes, especially the differentially responding IL10 and HSPA6 genes, can help associate parasite markers with the host immune response.

**FIGURE 5:**
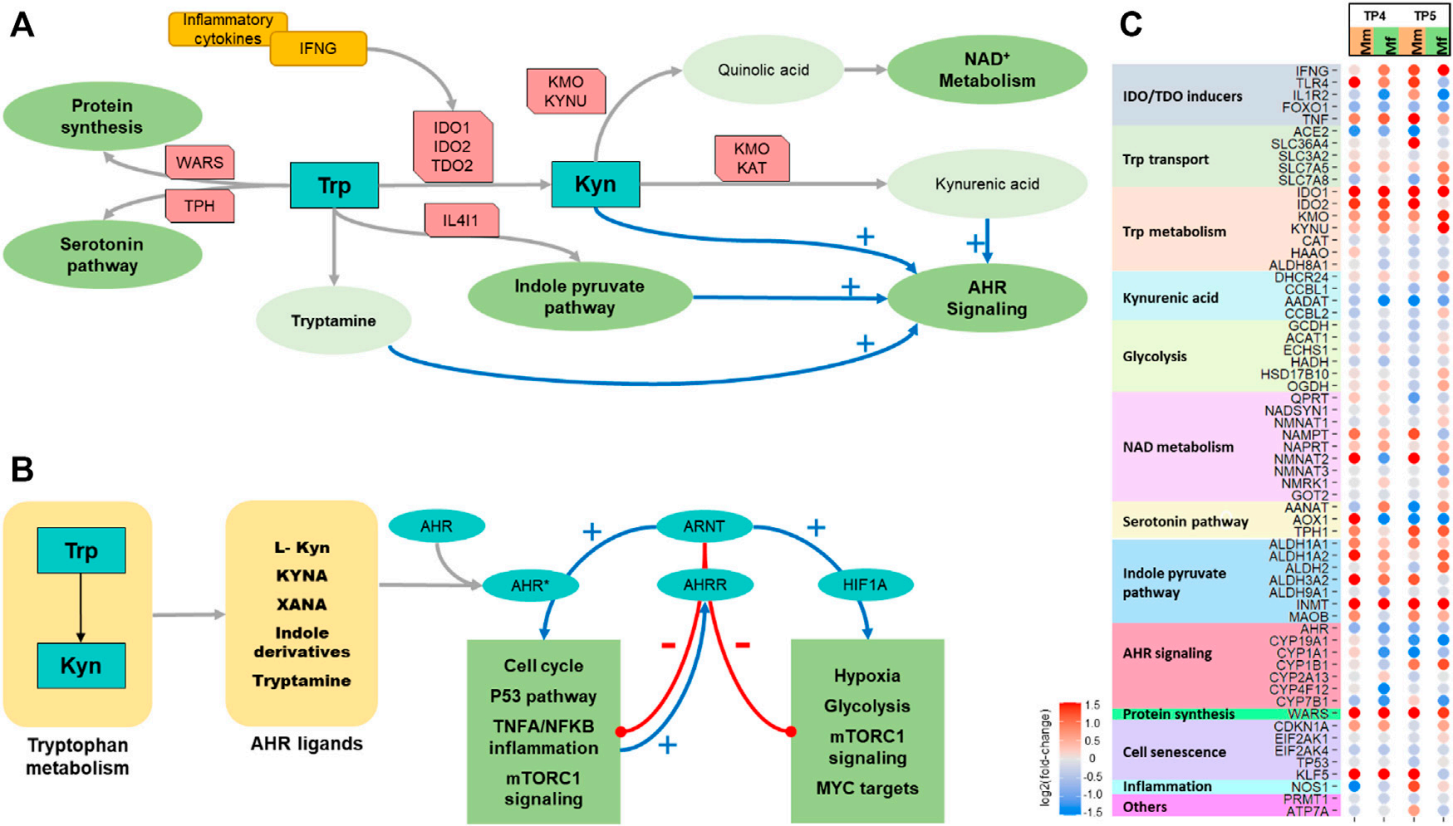
Tryptophan metabolism. **(A)** Schematic showing key features of tryptophan metabolism. **(B)** Schematic showing AHR signaling. **(C)** Heatmap of differential expression of significant genes involved in tryptophan metabolism comparing the two hosts across TP4 and TP5.

On the host side, differences in the mechanisms for pathogen detection and PRR signaling pathways are surprisingly subtle. However, these differences are magnified downstream with MAPK signaling. There is a close relation of these signaling cascades, especially the GPCR activity with the p53 pathway and cell cycle (Zhang and Liu, 2002; Goldsmith and Dhanasekaran, 2007; New and Wong, 2007). Ca^2+^ drives intracellular communication and interacts with GPCR to regulate various aspects of the cell cycle, and by extension, regulates inflammation and apoptosis during infection. This regulation is even further augmented by inflammasome activity (Figure 5). Specifically, some of the Ca^2+^ binding S100 proteins (S100A8, A9 and A4) might be differentiating factors between the two hosts. While S100A8 and S100A9 aid the inflammasome assembly, S100A4 assists with the regulation of the p53 pathway. Additionally, the inflammasome assembly process is regulated by multiple other factors including ROS, IL10 and transcription factor AP-1. These factors do not only relay the stress response but also seem to be important in regulating the p53 pathway.

The most strongly differentiating factor between the two species appears to be the control of cell proliferation by Mf during log phase via the p53 pathway, along with subsequent inhibition that leads to recovery. Similar stresses can trigger both inflammation and cell proliferation, but it appears that it is the stress related to fundamental ribosomal assembly that causes the inhibition of cell proliferation in Mf through the p53 pathway. Several upstream kinases have been shown to cause this stress. Since ribosomal assembly is one of the most energy intensive functions, inhibition of this fundamental function to conserve energy seems likely (Albert et al., 2019). Of course, that is not the sole purpose. In particular, we observe that ribosomal assembly leads to differences in p53 based cell cycle arrest and DNA repair in Mf. The interrelatedness of this pathway with p21, AP-1 and HSF1 activity provides additional regulators that might be responsible for balancing cell proliferation with inflammation.

Another known inflammation biomarker, the Kyn/Trp ratio, shows surprisingly deep integration with these processes. Even though the induction of IDO in malarial infection is quite often discussed (Sanni et al., 1998; Hansen et al., 2000; Tetsutani et al., 2007; Colvin and Joice Cordy, 2020; Santos et al., 2020), its biological significance for the immune response is in general poorly understood. Nonetheless, a mathematical model of the direct upregulation of IDO through IFNγ signaling quite clearly shows how the Kyn/Trp ratio changes during the infection (Figure 5). This metabolic model is able to shed light on several important, although indirect implications, such as the importance of KMO and KYNU in regulating fluxes, redirection of fluxes towards NAD^+^ metabolism, and metabolite pools of kynurenine compounds as ligands for AhR. In summary, Trp metabolism diverts the fluxes towards the essential functions, and especially NAD^+^ metabolism and protein synthesis. The higher activity in Mf also indicates that this host maintains essential functions in spite of the inflammation. Further analysis into the kynurenines shows an impactful control of AhRR in regulating both AhR and HIF1α related signaling. This process includes a competitive effect of multiple stresses, hypoxia and infection induced damage and cytokine response in determining the overall outcome.

Although this analysis dives deep into multiple molecular mechanisms that play crucial roles in permitting resilience of the host, it only paints a crude image of the immune response over time. For example, a more detailed longitudinal and immunologically based analysis of *SICAvar* gene expression and switching of SICA proteins in each host (and with different parasite species (Cordy et al., 2019)) is likely to advance our understanding of the different antibody responses and immune evasion mechanisms (reviewed in Galinski et al. (2018)). The combined analysis of immune response, inflammation and cell proliferation also seems to reveal Ca^2+^ as a crucial factor, which is known to play a role in iRBC egress (Glushakova et al., 2013). If this general finding can be validated and cross referenced with other bacterial and viral infections (Tran Van Nhieu et al., 2018; Chen et al., 2019; Crespi and Alcock, 2021), improved understanding of Ca^2+^ homeostasis might lead to novel targets that could naturally aid the immune response against *Plasmodium* infection. Similarly, the metabolic model we employed, adjusted for transcriptional changes during the infection, provides a deeper appreciation of the mechanisms of Trp metabolism and could possibly be extended to identify targets that could predictably adjust metabolism to aid in resilience.

Overall, this work interprets transcriptional data and integrates them in a manner that provides deeper understanding of *Plasmodium* infections. It is hoped to suggest new avenues of studying malaria and identifying valid candidates for future drug development.

## Methods

### Experimental Setup and Data Pre-Processing (Ribonucleic Acid Seq/LC-MS)

The analysis described here expands on previously published studies (Gupta et al., 2021) with details about individual processes. Briefly, four male Mm and seven male Mf were infected with *P. knowlesi* sporozoites. Peripheral blood samples were extracted before (baseline) and after inoculation with sporozoites (pre-patent—TP3, log-phase—TP4 and peak-phase—TP5). These blood samples were used for transcriptomics and metabolomics analysis (Supplementary Figure S1).

To assess the transcriptome, samples were sequenced using Illumina Hi-seq 3,000, mapped using STAR and normalized using DESeq2. Details of the process were previously published in Gupta et al. (2021).

For metabolomics analyses, plasma samples were quantified using the AbsoluteIDQ p180 kit (Biocrates Life Sciences AG). Specifically, the metabolites were quantified using SCIEX Exion LC and a QTRAP 5500 mass spectrometer in only positive ionization mode with each sample injected using a separation column. Specific details of the process can be found with the corresponding submissions of MaHPIC data to PlasmoDB (https://plasmodb.org/plasmo/app/static-content/PlasmoDB/mahpic.html) with the Mm dataset available at MTBLS824 and the Mf dataset at MTBLS822 from the MetaboLights repository.

### Enrichment Analyses

Differential expression (DE) of genes was calculated using DESeq2. Genes with low read counts were removed from analysis. The genes were modeled using the design—*Species + TimePoint + Species:TimePoint* and DE was calculated using Wald’s test.

Gene set enrichment analysis was performed using the GSEA toolkit (version 4.0) of the Broad Institute. The gene sets used for the analysis were Hallmark (Liberzon et al., 2015), Reactome (Jassal et al., 2020), ImmuneSigDB (Godec et al., 2016) and Gene Ontology (Ashburner et al., 2000; Gene, 2021). The pre-ranked GSEA module of the toolkit (Subramanian et al., 2005) was used for the analysis, and all genes were ranked based on inverse of adjusted *p*-values and the sign of fold changes. Files of custom gene sets (gmt files) were created using R to contrast enrichment scores between comparable data sets. To compare gene sets across the two species and account for representation bias in individual gene sets, rank scores for all genes were used to calculate enrichment scores (ES), which were adjusted by normalization of gene set sizes. Gene sets with small (<15) and large (>500) overlaps were filtered out. This normalized enrichment score (NES) was used to contrast various gene sets.

Enrichment analysis for targets of AhR, AHRR and HIF1A was performed similarly to the method described above. The gene sets for target genes for each were created using ChIP-Atlas (Oki et al., 2018) with ±5 Kb overlap with the transcription start site. NES values for each subset described in the *Results* were calculated with the method described above.

### Weighted Gene Co-Expression Network Analysis

Weighted gene co-expression network analysis (WGCNA) was performed using the WGCNA package (version 1.70–3) (Zhang and Horvath, 2005; Langfelder and Horvath, 2008) in R to describe correlation patterns among genes. The analysis was performed in multiple ways to serve different purposes. The differences arose in the subsets of samples in datasets used for each analysis. First, for co-expression networks with both host and pathogen genes, only infection TPs (TP4 and TP5) were used for both hosts, as there are no pathogen transcripts at baseline and TP3. Next, to differentiate host-specific differences, subsets of each host for different infection TPs were used. Finally, all TPs for both hosts were used with host-only genes to form co-expression networks among host genes.

WGCNA analysis begins with creation of a Pearson correlation matrix of the expression of all gene pairs. These were used to filter highest correlated pairs where required. This step was followed by the creation of an approximately scale-free adjacency matrix, using a power function. The soft threshold parameter (B) for the power function in each case was determined based on the criterion of approximate scale-free topology, as described in the software manual (Langfelder and Horvath, 2008). The topological overlap matrix (TOM) was calculated to quantify the degree of overlap in shared neighbors. Finally, modules were created using a dynamic tree cut algorithm in WGCNA. To characterize each of the modules, module eigengenes and GO annotations were calculated. To calculate the similarities between various modules, Pearson correlation between eigengene vectors was used.

### Deconvolution of Cell Populations

Cibersortx (Newman et al., 2019) was used to analyze gene expression data to obtain an estimation of abundances of individual cell types from mixed cell populations in the various blood samples. The LM22 signature matrix (Newman et al., 2015) was used as a cell type reference profile. Previously DESeq2 normalized expression data for all samples were used to estimate the abundances of the 22 cell types from whole blood.

To contrast various groups, the lmFit function (limma package) in R was used to model the cell populations as *Species + TimePoint + Species:TimePoint* and the eBayes function was used to compute log fold changes, *t* statistics, *p*-values and adjusted *p*-values, using the Benjamini–Hochberg method.

### Dynamic Modeling of Tryptophan Metabolism

To understand the implications of transcriptomic changes during *P. knowlesi* infection, we used a well-established tryptophan metabolic model (Stavrum et al., 2013) and adjusted its parameters to represent changes in enzymatic activities in accordance to changes in the expression of corresponding genes (Tang et al., 2018).

The model was originally developed for liver tissue and had to be adapted for blood. Due to the lack of tissue specific enzyme concentration data, we used gene expression data for individual tissues (in this case blood *vs* liver data from the GTEx project (Consortium, 2013)) to form a crude estimate of enzymatic concentration. Each reaction rate v in the model is described with the Michaelis-Menten rate function

$$v=\frac{V_{\text{max}}.\;S}{K_{m}+\;S}\;$$ (1)

where $V_{\text{max}}$ is the maximum reaction rate, S is the substrate concentration and $K_{m}$ is the Michaelis constant. According to our assumption of proportionality between gene expression and enzyme activity (Tang et al., 2018), $V_{\text{max}}$ is a function of enzyme concentration and enzymatic turnover $K_{cat}$ . Since enzyme concentration is difficult to calculate, mRNA levels were used as approximate quantities:

$$V_{\text{max}}=F.\;mRNA.\;K_{cat}$$ (2)

Here, F is a factor that converts expression values into enzyme concentrations and mRNA is the measured expression.

Once the parameters were updated, the model was simulated to a steady state to obtain baseline metabolite concentrations and fluxes for the blood model.

Next, the kinetic parameters were updated by a factor corresponding to the fold change in gene expression in order to obtain the appropriate enzymatic activity, similar to Eqs. 1 and 2. For each case, the model was simulated to the steady state of all metabolite concentrations and fluxes were used for comparison of different scenarios.

For flux control analysis (Wildermuth, 2000) (Eq. 3), the control coefficients were calculated as

$$C_{v_{i}}^{S}=\;\frac{d\ln J}{d\;ln\;v_{i}}$$ (3)

where $C_{v_{i}}^{S}$ is the flux control coefficient for the pathway flux J with small changes in enzyme activity $\;v_{i}$ of step i .

## Data Availability

Only publicly available datasets were analyzed in this study. They can be found at https://plasmodb.org/plasmo/app/static-content/PlasmoDB/mahpic.html (Experiments 06 and 07) or under BioProject accession PRJNA524357 (https://www.ncbi.nlm.nih.gov/bioproject/524357) and PRJNA526495 (https://www.ncbi.nlm.nih.gov/bioproject/526495).
