# Supplementary material for: Dynamic Control Balancing Cell Proliferation and Inflammation is Crucial for an Effective Immune Response to Malaria
Source: Front Mol Biosci. 2022 Feb 15;8:800721. doi: 10.3389/fmolb.2021.800721 (PMC8886244; doi:10.3389/fmolb.2021.800721)
Supplement: Supplementary file 1 [file DataSheet1.docx]

**Supplements:**

Dynamic control balancing cell proliferation and inflammation is crucial for an effective immune response to malaria

**Anuj Gupta, Mary R. Galinski, Eberhard O. Voit**

These supplements contain additional figures and tables, as cited in the main text.

## Supplementary Figures


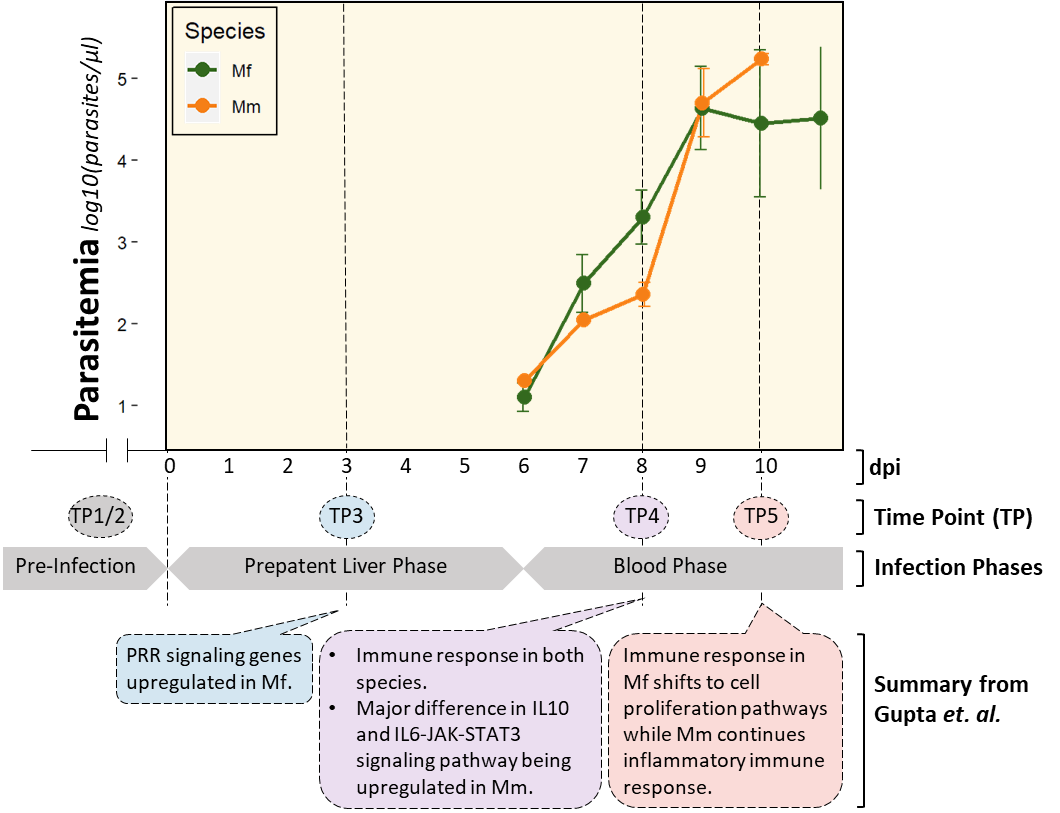


Fig S1. Timeline and progression of P. knowlesi infection in Mm and Mf. The figure shows the levels of parasitemia and corresponding time-points and days post infection on X-axis. The x axis also contains various infection phases and key observations from the previous transcriptomics study (Gupta et al., 2021).


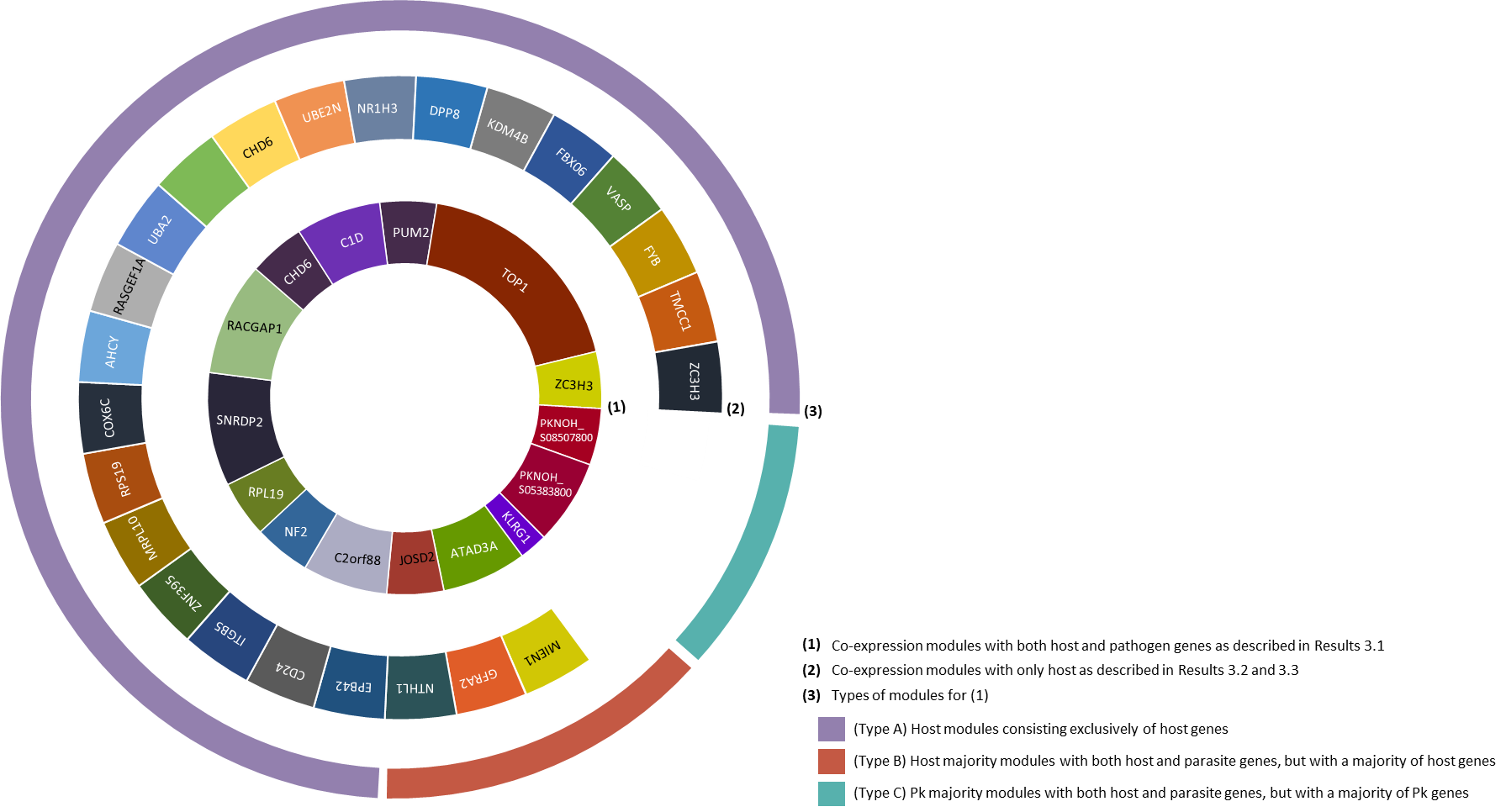


Fig S2. Illustration for WGCNA showing different analyses and their corresponding modules. Modules in (1) and (2) have been radially adjusted to be closest to modules most similar in the two analyses. (1) is corresponding to analysis for Sec 3.1. (2) is corresponding to the analysis for Sec 3.2 and 3.3.


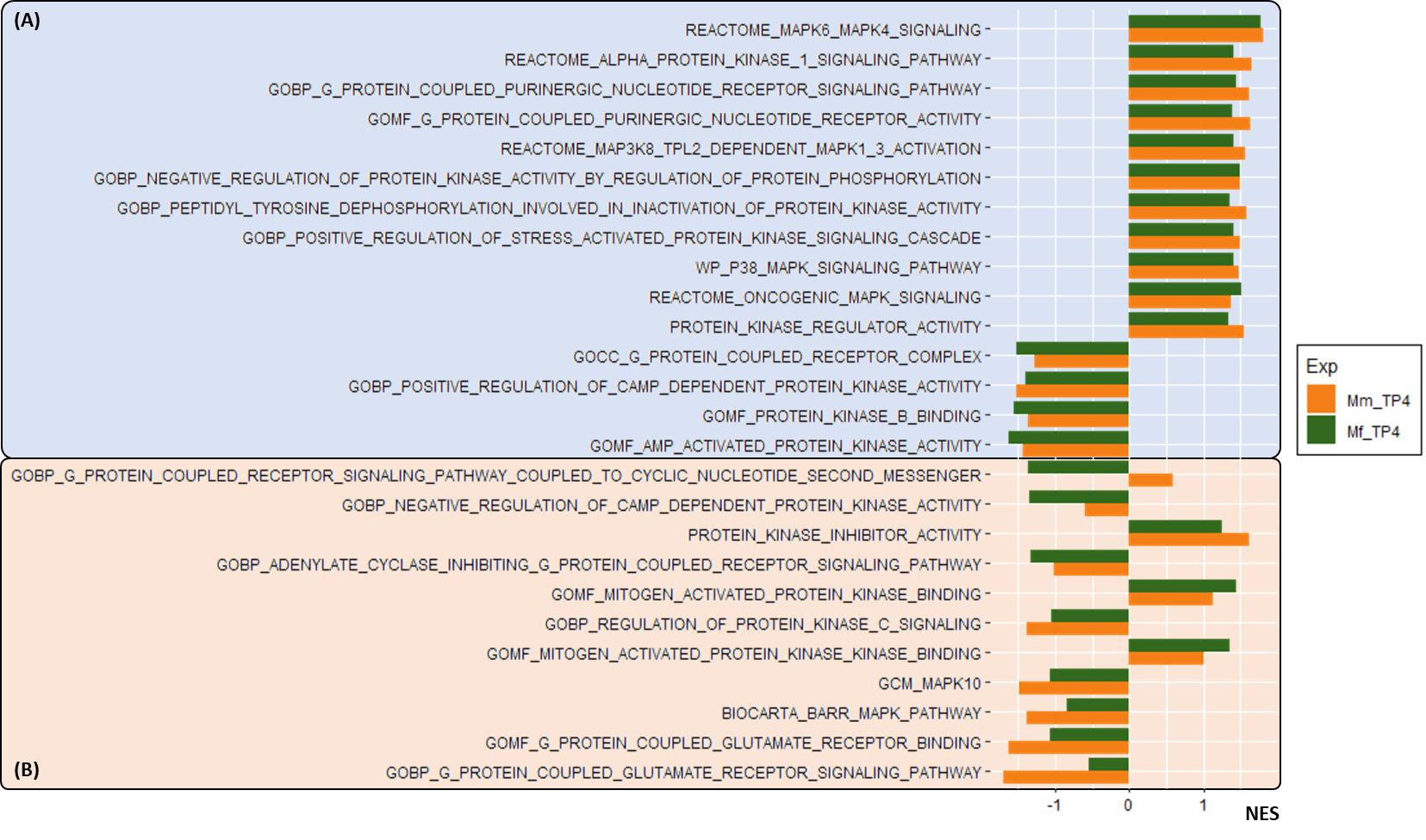


Fig S3. Bar plot for enrichment of gene sets related to protein kinase signaling. (A) Gene sets that are similarly enriched in both Mm and Mf during log phase (TP4). (B) Gene sets that are differently enriched in Mm and Mf during log phase (TP4).


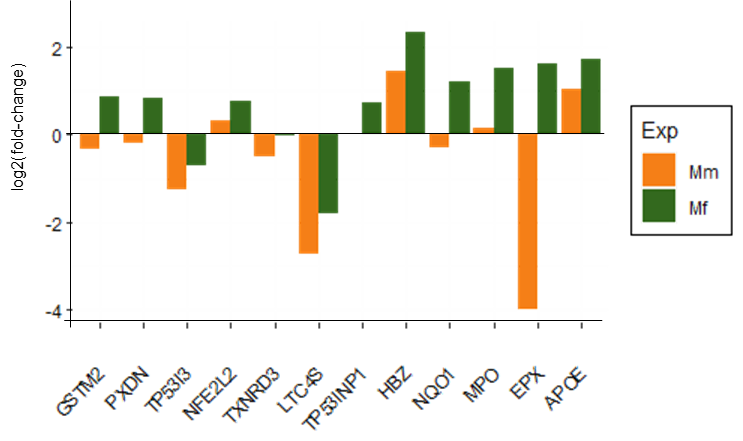


Fig S4. Bar plot of fold changes in the expression (at TP4) of genes involved in protection against ROS through antioxidants. These genes have also been shown to interact with the p53 pathway.


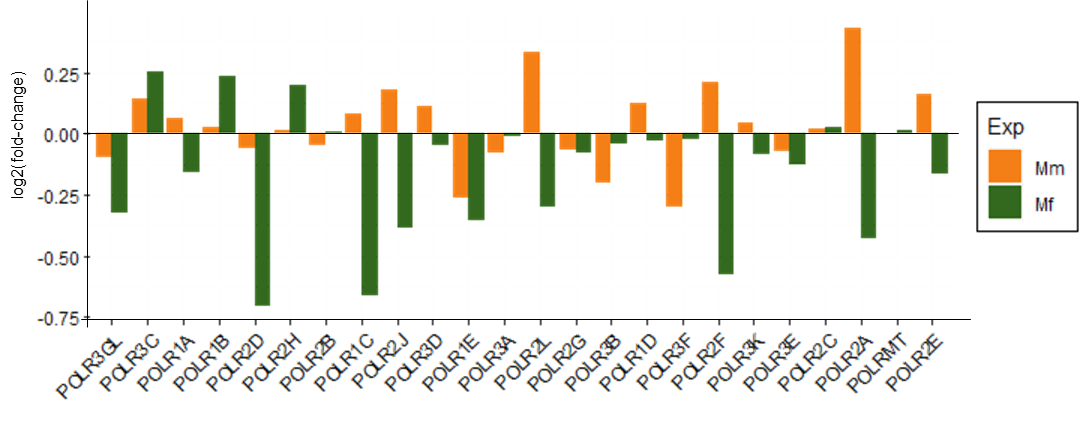


Fig S5. Bar plot of fold changes in the expression of RNA polymerase associated genes (at TP4) that are responsible for ribosomal biosynthesis. Worth noting are PLOR1C, POLR2E, POLR2A, POLR2J and POLR2L, as they correspond to downregulation of ribosomal biosynthesis in Mf at TP4.


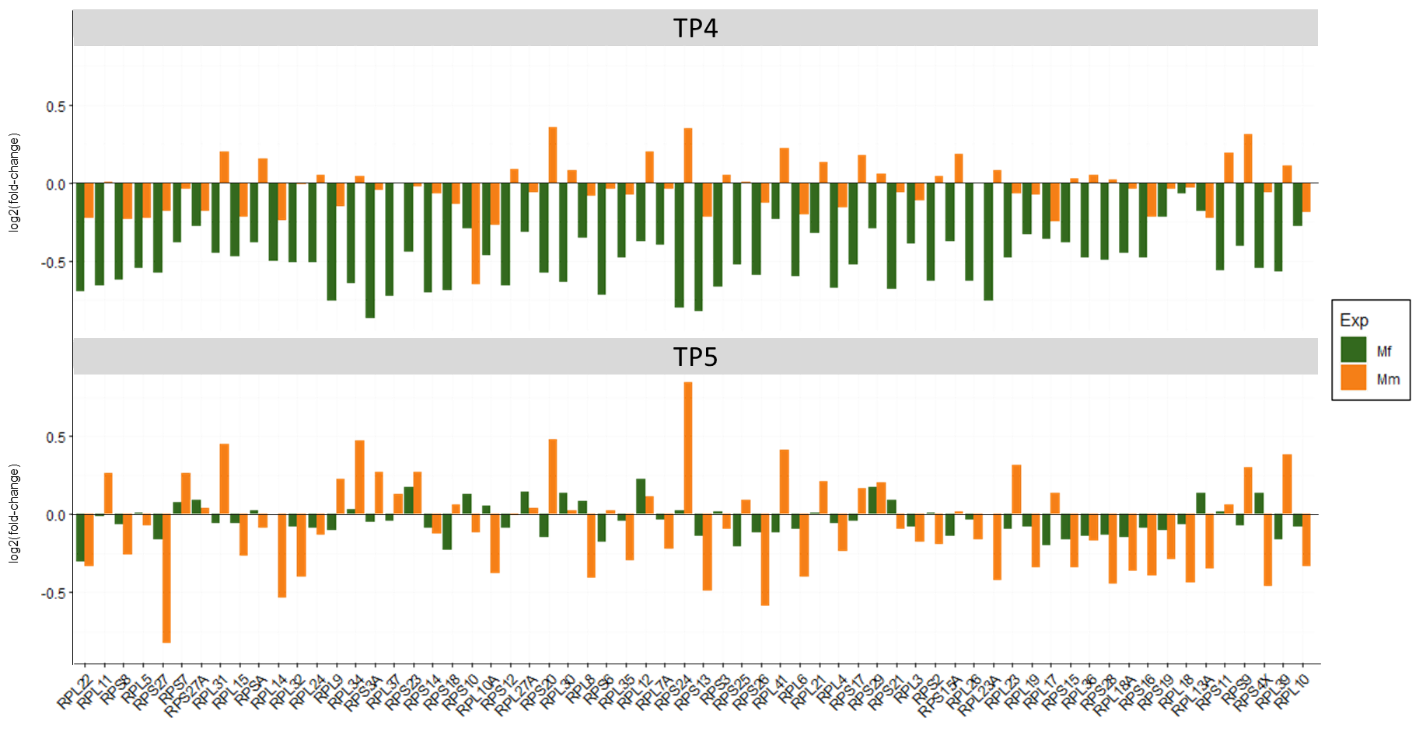


Fig S6. Bar plot of fold changes in gene expression of Ribosomal Proteins (RPs), comparing both hosts across TP4 and TP5. This comparison highlights the differences between TP4 and TP5.


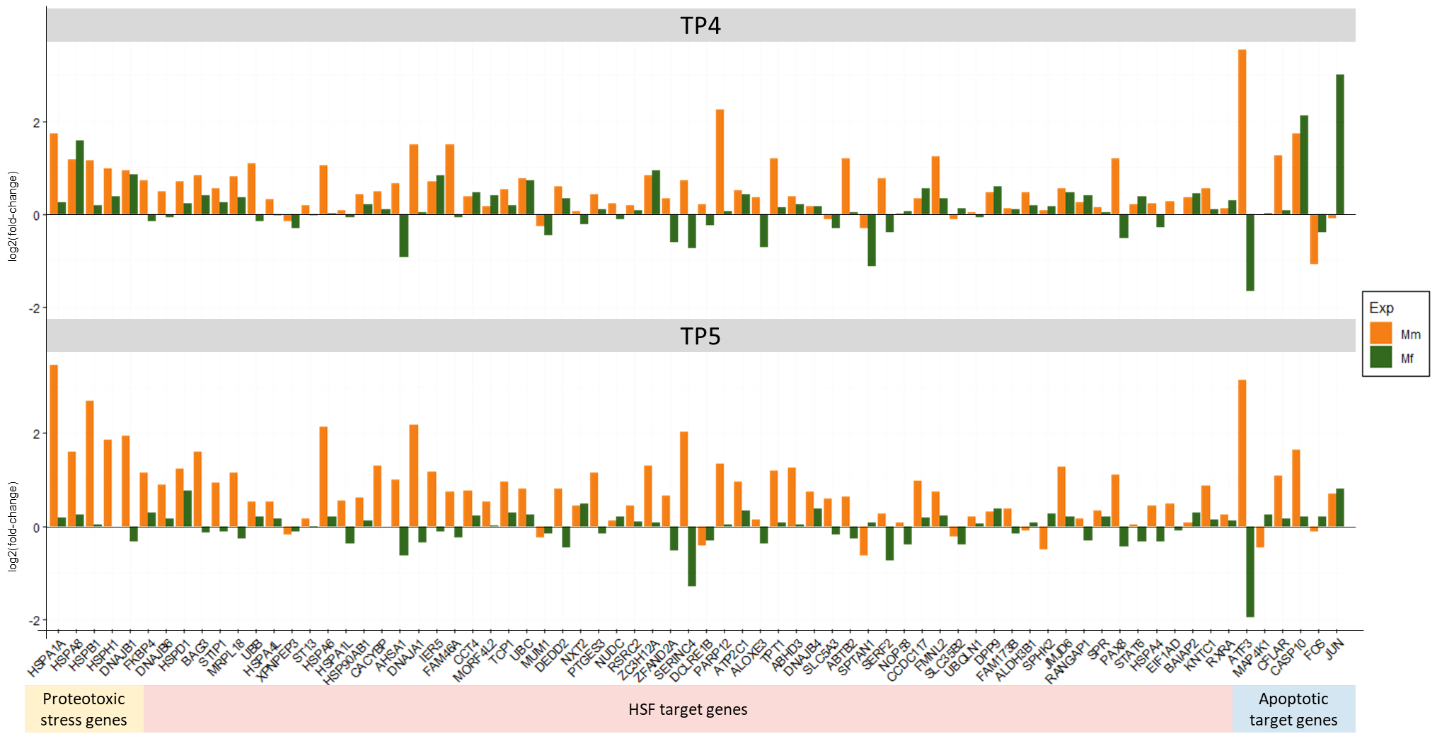


Fig S7. Bar plot of fold changes in the expression of genes coding for heat shock factor (HSF) related proteins (chaperones) and signals for stress, Hsf1 and apoptotic target genes, comparing Mm and Mf across TP4 and TP5.


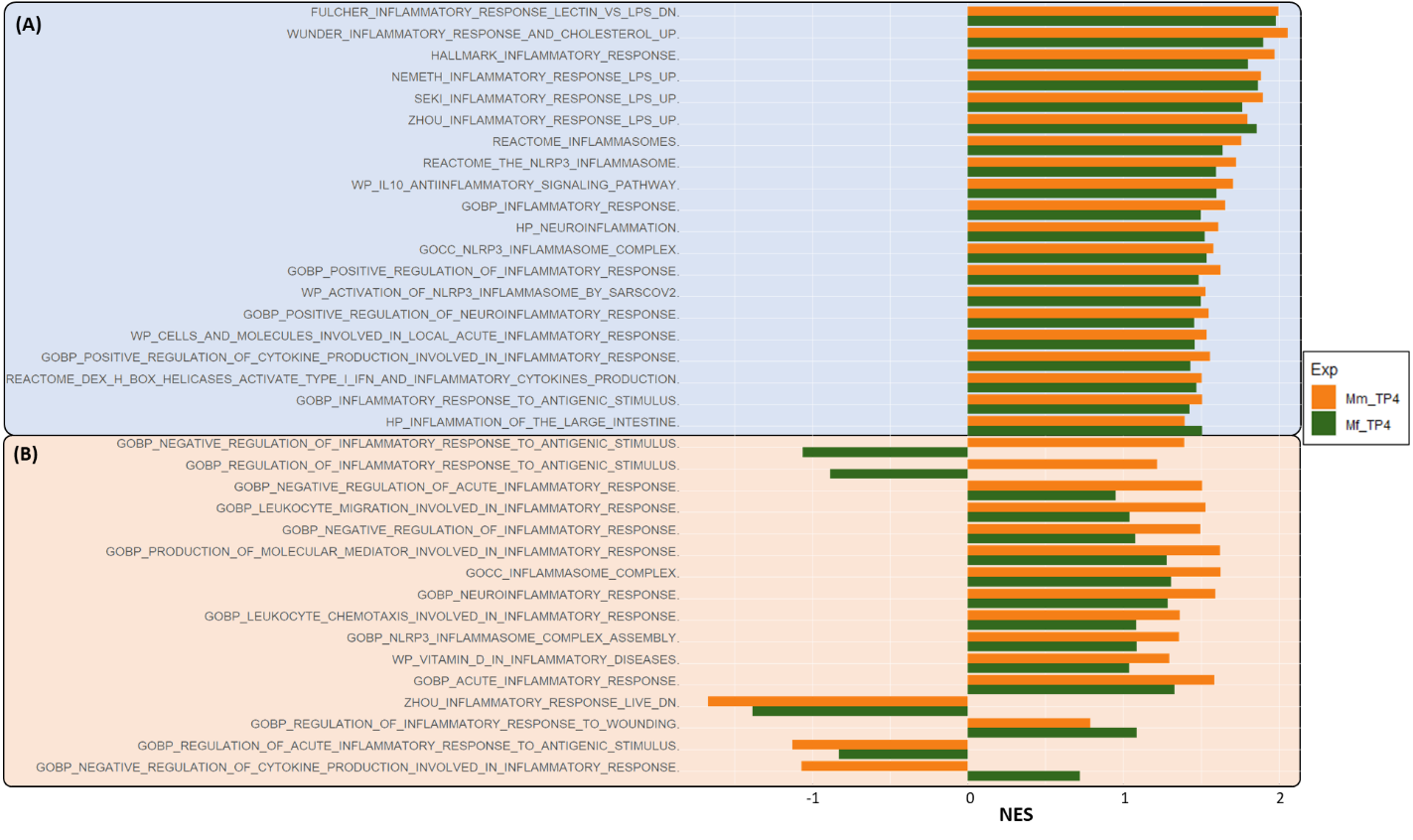


Fig S8. Bar plot of enrichment scores of inflammatory gene sets during log phase of infection (TP4). The plot highlights (A) similar (light blue) and (B) distinguishing (light orange) gene sets between Mm and Mf.

*
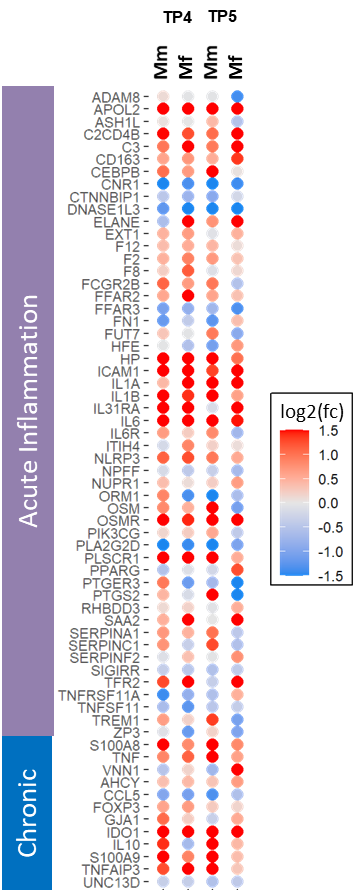
*

Fig S9. Heatmap showing various inflammatory genes related with acute and chronic inflammation for the two hosts.


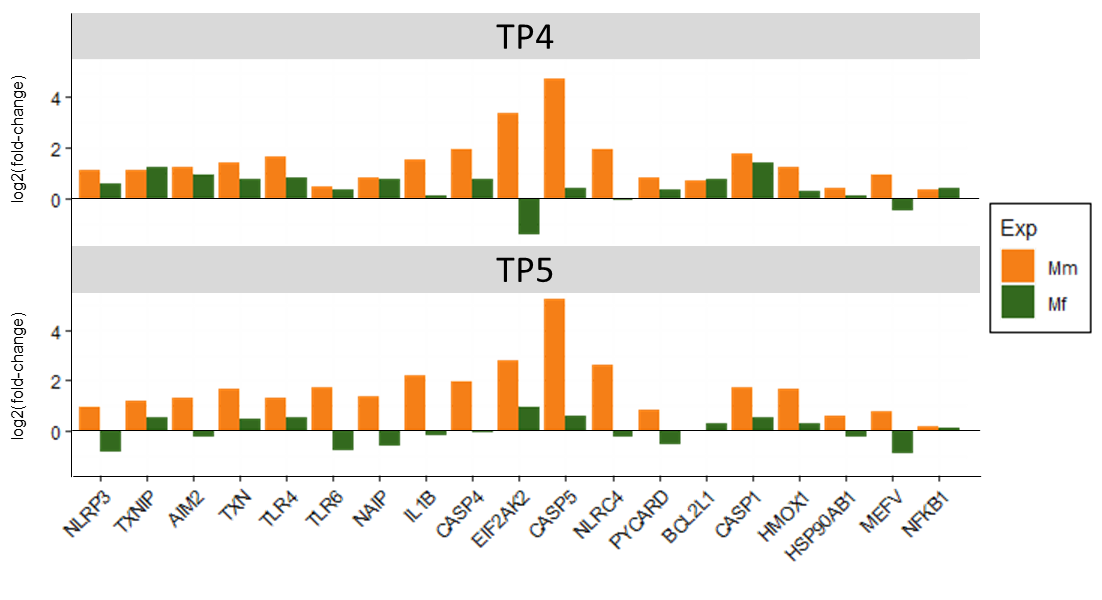


Fig S10. Bar plot of fold changes in the expression of genes involved in the inflammasome complex and its regulation across TP4 and TP5, differentiating Mm from Mf.


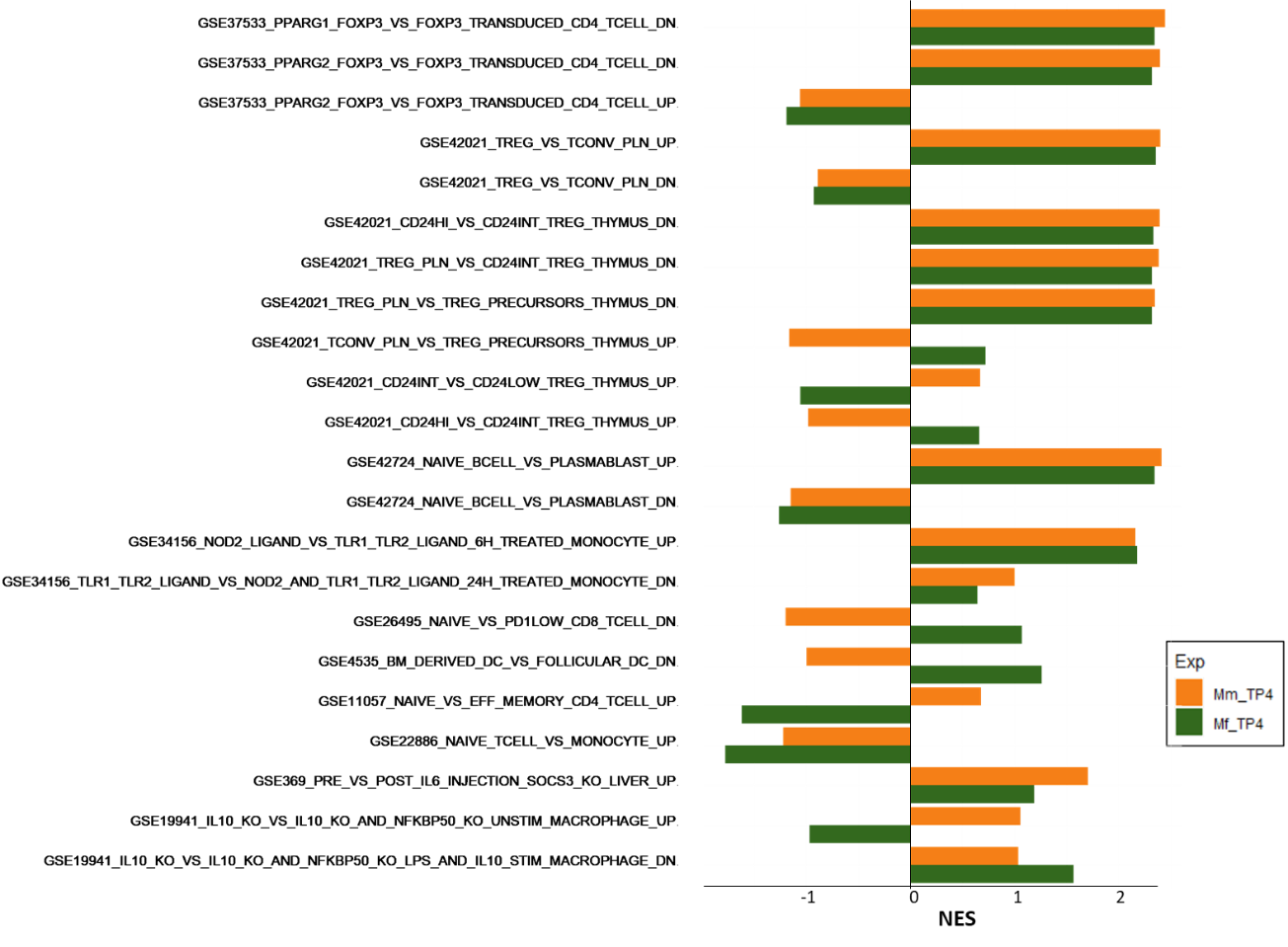


Fig S11. Bar plot of enrichment scores of important immunologic signatures. The plot shows similarities and differences between Mm and Mf during log phase.


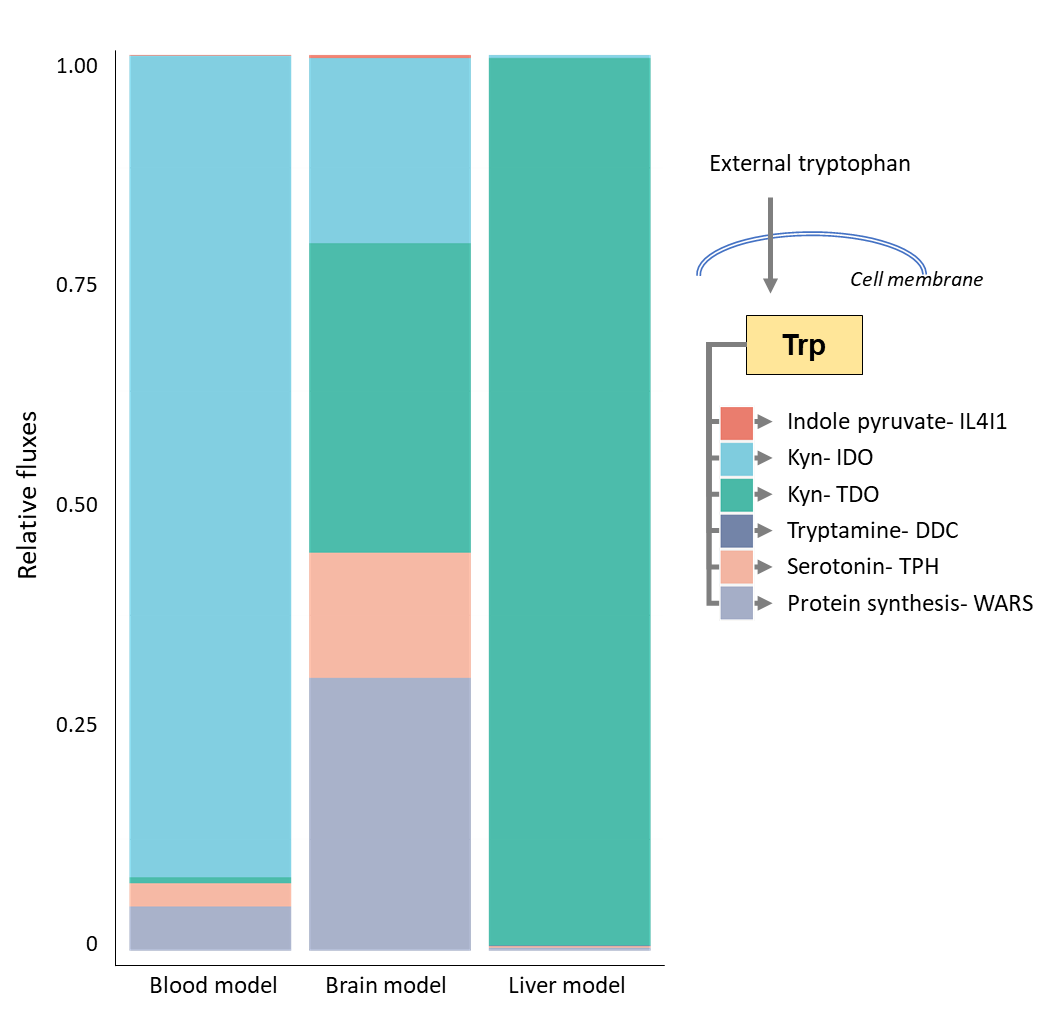


Fig S12. Comparison of flux distributions through Trp in blood, brain and liver models.


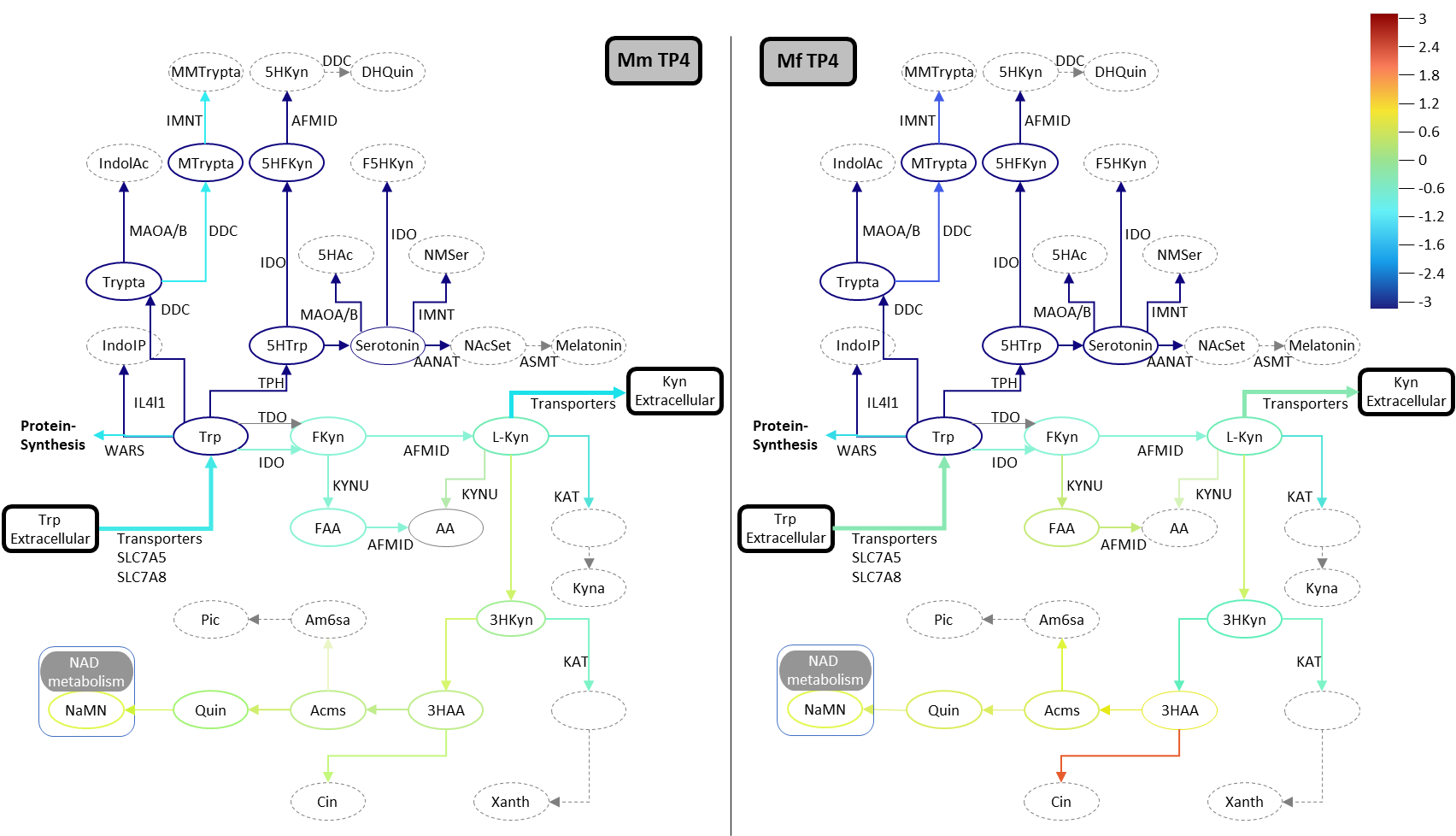


Fig S13. Tryptophan metabolism model adjusted for enzymatic activity for Mm and Mf at TP4. The colored arrows show corresponding changes in fluxes (log2 fold change) while colored ovals show corresponding changes in metabolite concentrations (log2 fold change) in comparison to the baseline as predicted by the model.


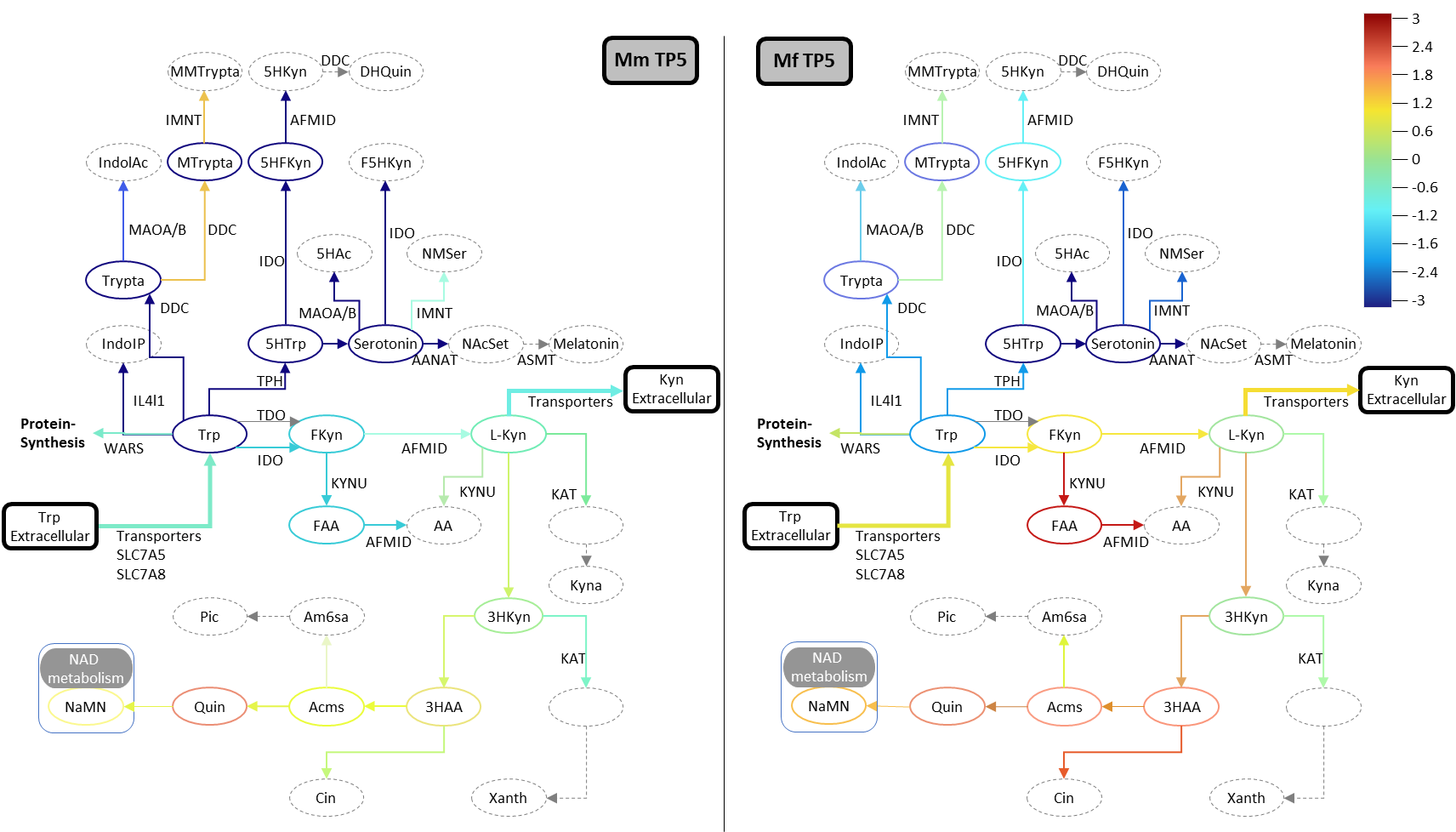


Fig S14. Tryptophan metabolism model adjusted for enzymatic activity for Mm and Mf at TP5. The colored arrows show corresponding changes in fluxes (log2 fold change) while colored ovals show corresponding changes in metabolite concentrations (log2 fold change) in comparison to the baseline as predicted by the model.


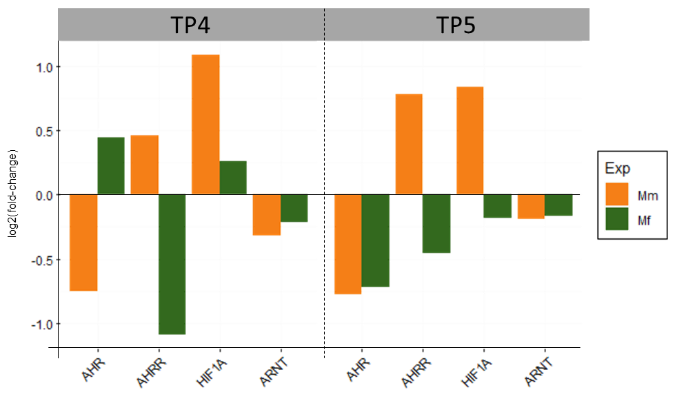


Fig S15. Changes in bHLH-PAS superfamily genes involved in AhR signaling.


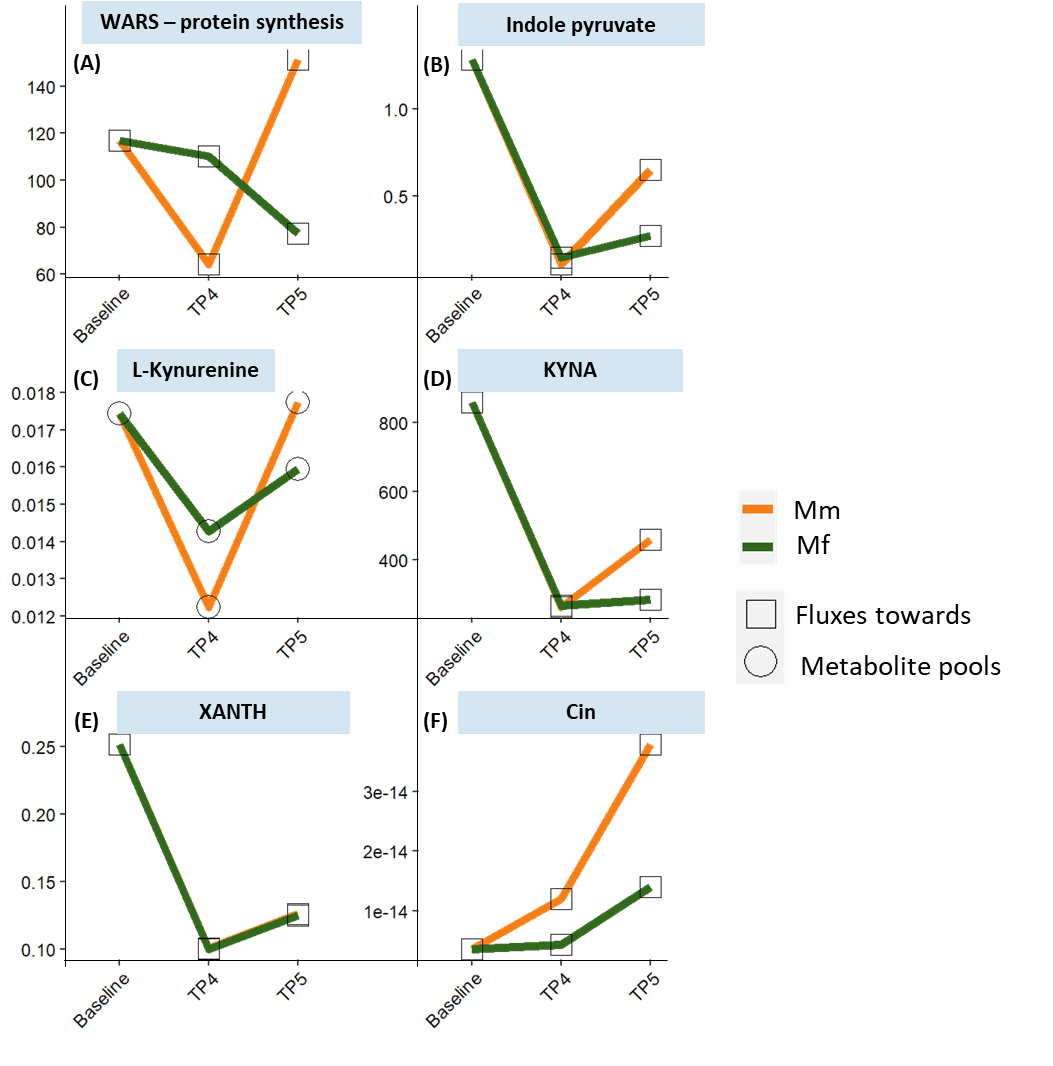


Fig S16. Model predictions for metabolites and fluxes during infection. (A) WARS driven protein synthesis of tryptophan. (B-F) Tryptophan metabolism derived ligands for AhR measurements (fluxes and metabolite pool) as observed from the model. (B) Tryptophan derived indole pyruvate ligand for AhR. (C-F) Kynurenine derived kynurenines as ligands for AhR.


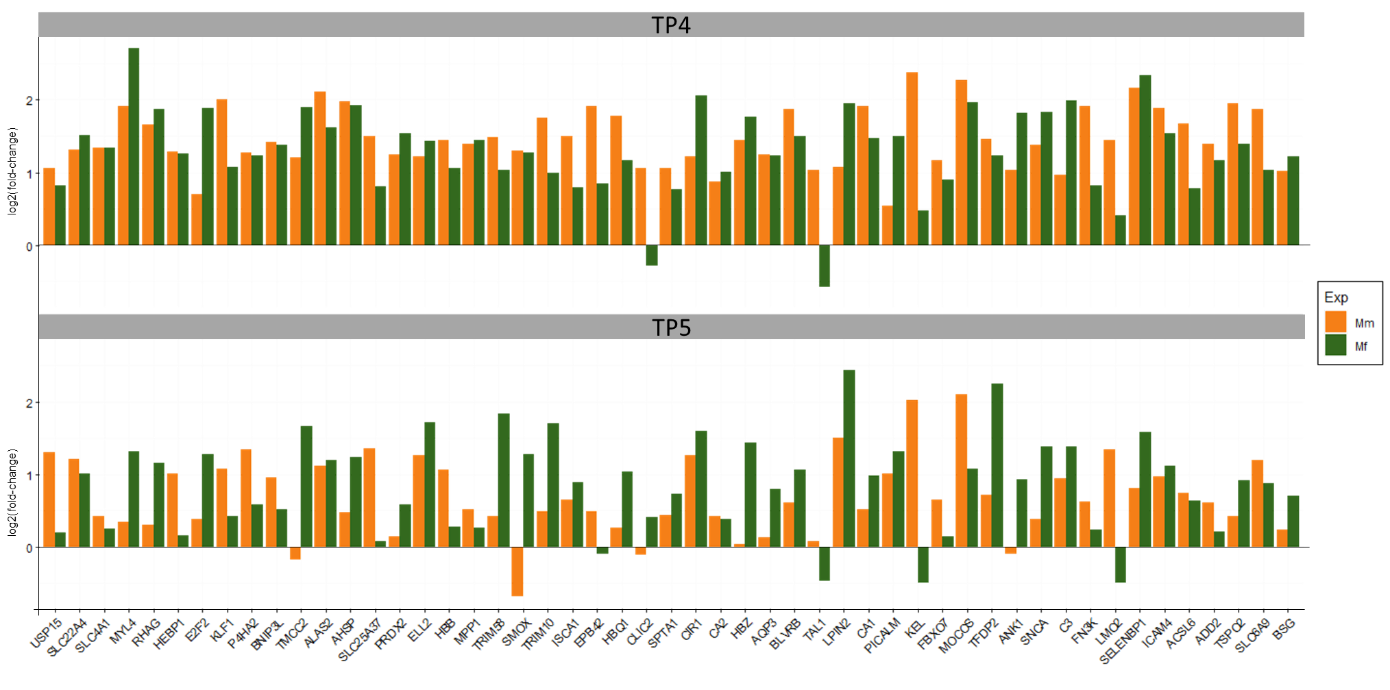


Fig S17. Bar plots of fold changes in expression of genes related to of heme metabolism comparing the two hosts across TP4 and TP5.


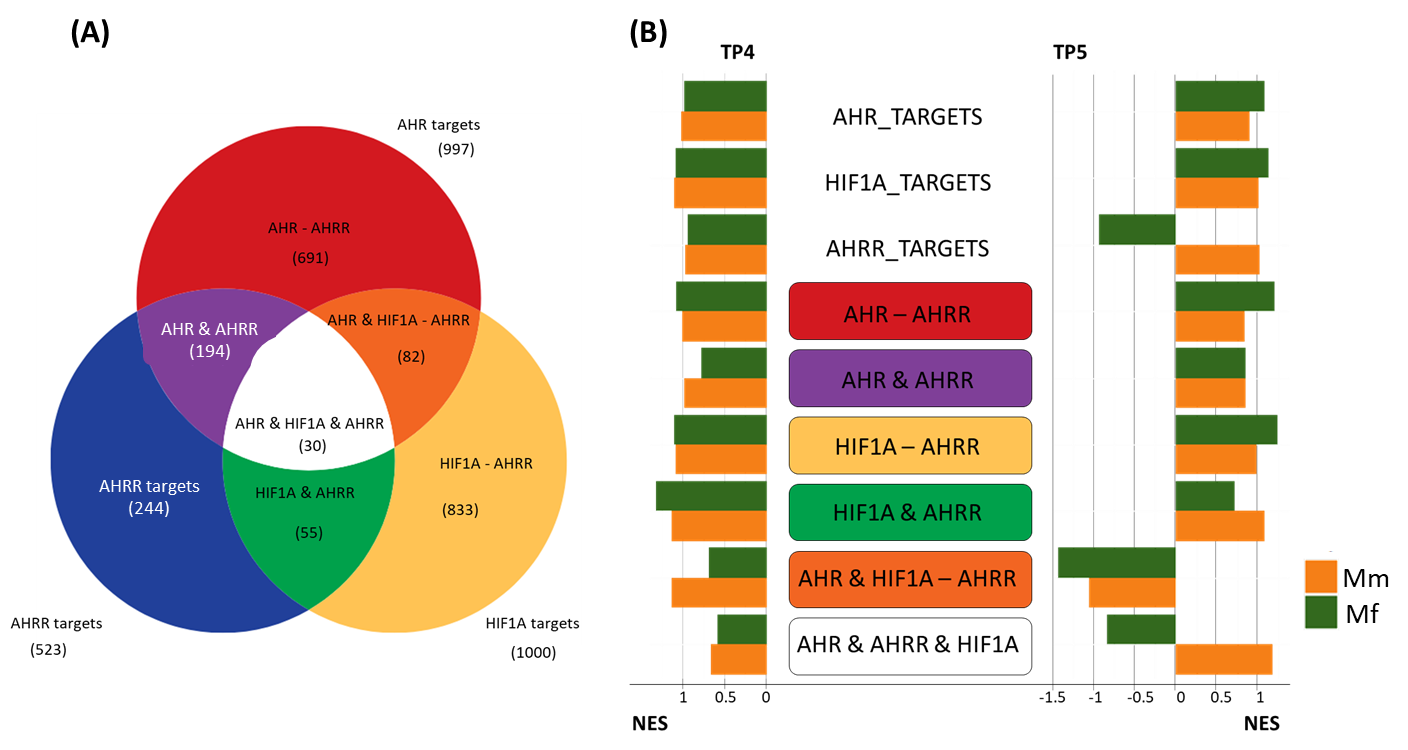


Fig S18. Results regarding AhR, AhRR, ARNT and HIF1α complexes. (A) Venn diagram for targets for the three complexes involving the aryl hydrocarbon receptor nuclear translocator (ARNT): AhR:ARNT, AhRR:ARNT and HIF1α:ARNT. (B) Enrichment of target genes for members of the bHLH-PAS superfamily of transcription factors during TP4 and TP5. Enrichment of AhR (red) and HIF1α (beige) target genes highlighting the effect of AHRR (TP4). AHR\AHRR: AhR-only targets not affected by AhRR; AHR & AHRR: AHR and AHRR targets; HIF1A\AHRR: HIF1A-only targets not affected by AhRR; HIF1A & AHRR: HIF1A and AHRR targets; HIF1A & AHR\AHRR: HIF1A and AHR targets not affected by AhRR; AHR & AHRR & HIF1A: Targets for HIF1A, AHR and AHRR. Here, “&” represents presences of both components, while “\” represents absence of the second component.


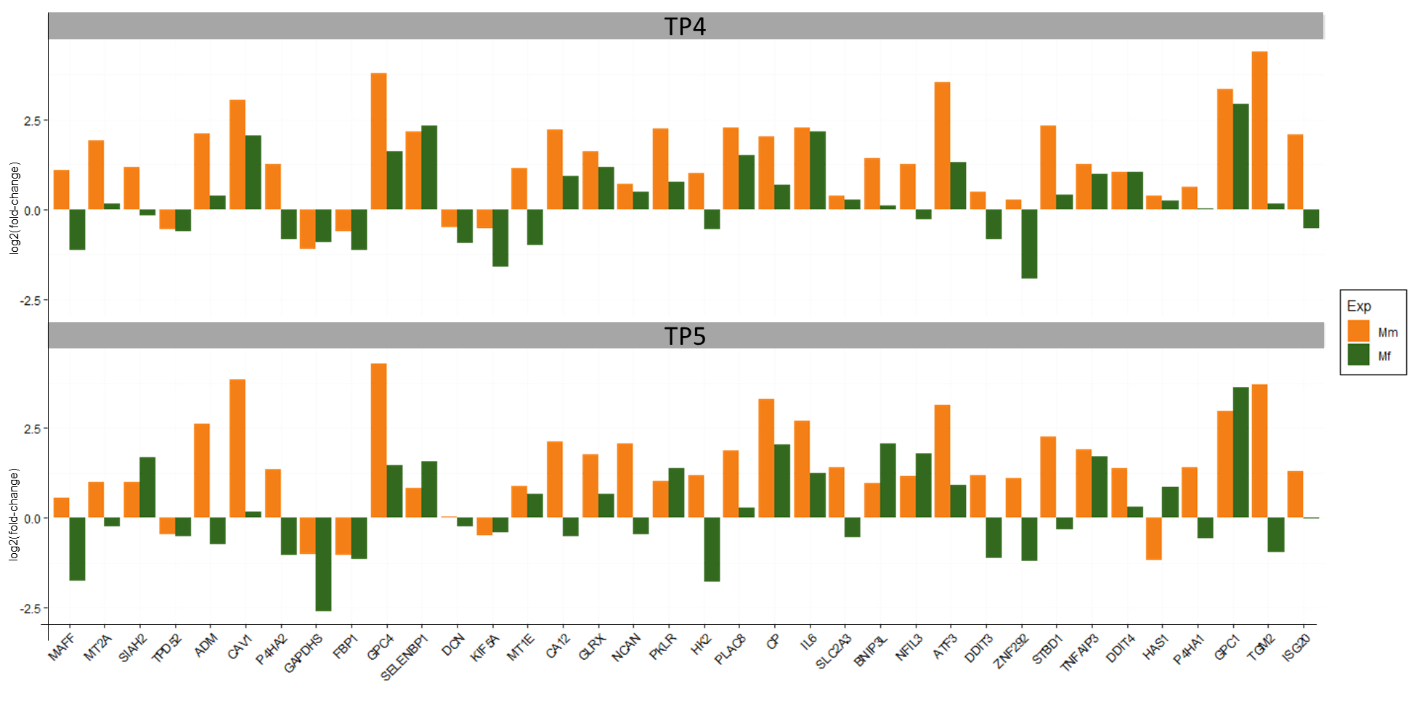


Fig S19. Bar plot of fold changes of hypoxia related genes compared between the two hosts across TP4 and TP5.

## Supplementary Tables

Table S1(A): Co-expression modules obtained from WGCNA and their functional annotation (with Pk genes and excluding TP1,2,3) corresponding to Sec 3.1.1 (Fig S2(1)).

Table S1(B): Host-pathogen transcript pairs with significantly high correlation (r>0.7; FDR p <0.01). Most of the host and pathogen genes belong to modules ATAD3A and PKNOH_S08507800.

Table S1(C): Blast results showing gene names and accession ids for P. knowlesi transcripts that are highly correlated to host genes. The majority are SICAvar genes.

Table S2: Co-expression modules obtained from WGCNA and their functional annotation (without Pk genes and including TP1,2,3) corresponding to Sec 3.1.2 (Fig S2(2))

Table S3(A): Functional Annotation (DAVID^53^) of differentially expressed PRR genes in Mm and Mf showing specific aspects of PRR signaling pathway activated during log phase of infection

Table S3(B): Key differentially expressed PRR signaling pathway genes (DEGs) in Mm and Mf and differentially responding genes (DRGs) in Mm vs. Mf during the log phase of infection.

| DEGs in Mm and Mf | | | |  | DRGs: Mm vs. Mf | | |
| --- | --- | --- | --- | --- | --- | --- | --- |
| CTSK | RIPK2 | TLR3 | ITGAM |  | BIRC2 | NLRP6 | UBA52 |
| RSAD2 | DDX58 | TICAM2 | NOD2 |  | CAV1 | NR1H3 | UBE2D1 |
| NMI | TLR4 | IRF1 | NR1D1 |  | CD300A | PELI1 | USP15 |
| TANK | UBE2D1 | HAVCR2 | DHX58 |  | CD36 | RPS27A |  |
| IFIH1 | PIK3AP1 | FLOT1 | IFI35 |  | EPG5 | SLC15A3 |  |
| TIFA | IRF7 | TNFAIP3 | CD300A |  | FFAR2 | SLC15A4 |  |
| ALPK1 | TBK1 | CAV1 | RIOK3 |  | IRAK2 | SLC46A2 |  |
| TLR2 | UBC | LY96 | TICAM1 |  | LACC1 | TLR5 |  |
| DDX60 | NFKBIA | XIAP | LILRA2 |  | LTF | TNIP3 |  |

Table S4 (Left): Reactome^117^ pathways enriched by PRR related DEGs in Mm during log phase. (Right): Reactome pathways enriched by PRR related DEGs in Mf during log phase. The contraposition highlights the subtle differences between the PRR pathway of the two hosts along with key responsible genes.

Table S5: Cibersortx^124^ results for deconvolution of various cell populations and their comparison across various cases. For example, panels A and B list changes in cell populations during log phase (TP4) from baseline for Mm and Mf.

Table S6: Various ligands for AhR^92,93^

Table S7 (Left) Important target genes for AhR, HIF1α and both AhR &HIF1α reflecting the complexity of the mechanism and its outcome. (Right) List of target genes of AhR and HIF1α that affect important processes like the p53 pathway, heme metabolism, cell cycle and immune system responses, notably IFN-γ and NFκB signaling.

##

## Supplementary Notes (SN)

### Host-specific integration with parasite gene expression

Constructing co-expression networks from expression profiles of both hosts maximizes the power to detect co-regulated host and pathogen genes that are consistent with both hosts, but the approach is not likely to identify genes that differentiate the hosts. To identify *P. knowlesi* genes that might be important for individual hosts, co-expression networks were formed using individual host data (Tables SN1,SN2).

In Mm, it is not surprising that immune response and energy production related modules (RAB11A, CCL1 and COBRA1; Table SN1A) account for the most DEGs during the log phase. These modules are highly correlated with pathogen proteins that are crucial for *Plasmodium* development, such as the AP2 family[49] and Kinesin-5[50], along with protein trafficking proteins, such as v-SNARE Vti1p and vacuolar protein sorting-associated protein 18. The most significantly correlated pairs of host and pathogen genes (Table SN1B,C) include host genes IFNG, CXCL1 and CXCL6 and pathogen genes of *SICAvar* Type 1, as well as genes coding for calcium or potassium channel proteins.

In Mf, by contrast, the formed modules did not have significant functional annotation (Table SN2A). Most of the DEGs during the log phase were part of modules TMEM164 and GIMAP7. Both these modules consist of genes for multiple important functions, including immune response and metabolism, suggesting a close orchestration of these modules. *Plasmodium* proteins that are correlated include *SICAvar* Type 1 and FAD synthetase. Interestingly, the host genes most correlated with *P. knowlesi* genes (Table SN2B,C) are responsible for wound healing and coagulation. These platelet-related host proteins (PF4, GP1BB, GP5 and GP9) are highly correlated with proteins containing a *P. knowlesi* heme/steroid binding domain. Platelet-related wound healing proteins and their correlated *P. knowlesi* transcripts might be associated with increased resilience as well.

Table SN1(A): Co-expression modules obtained from WGCNA with their functional annotation and most correlated P. knowlesi proteins for modules of the host (Mm) (see Section 3.1.3 of the Text)

Table SN1(B): Blast results showing protein names and accession ids for P. knowlesi transcripts that are highly correlated to the host (Mm).

Table SN1(C): List of key host genes (Mm) which are most highly correlated with pathogen transcripts.

| **Genes** | | | | | |
| --- | --- | --- | --- | --- | --- |
| NEK6 | IL12B | TMEM33 | HMMR | SLC7A11 | DARS1 |
| PSMA6 | AUNIP | HBS1L | ORC5 | MGST2 | MME |
| PSMD14 | RMI1 | PAIP1 | CNEP1R1 | VEGFA | ITGB1BP1 |
| TGFA | RAD54B | DDX6 | CEP97 | ATP7A | FBXL13 |
| CENPE | SASS6 | CLN5 | CENPK | S100A12 | FBXO33 |
| BRCA2 | NUP43 | EIF5A2 | CDKN2B | IL15 | AMN1 |
| CCNA2 | GADD45B | CHAC2 | PHLDA1 | CXCL6 | ZYG11B |
| SGO2 | BIRC3 | MRPL42 | PBK | NT5E | LYVE1 |
| SPC25 | RGS2 | MRPL35 | SKA1 | IER3 | ERI1 |
| TACC1 | KLHL42 | MRPL33 | USP3 | CD160 | TRDMT1 |
| FBXL22 | PLD6 | SRP9 | IFNG | SLC38A2 | ACOT13 |

Table SN2(A): Co-expression modules obtained from WGCNA with their functional annotation and most correlated P. knowlesi proteins for modules of the host (Mf) (see Section 3.1.3 of the Text)

Table SN2(B): Blast results showing protein names and accession ids for P. knowlesi transcripts that are highly correlated to the host (Mf).

Table SN2(C): List of key host genes (Mf) that are most highly correlated with pathogen transcripts.
